# An Overview of Stress Analysis Based on Physiological Signals: Systematic Review of Open Datasets and Current Trends

**DOI:** 10.3390/s25237108

**Published:** 2025-11-21

**Authors:** Chariklia Chatzaki, Manolis Tsiknakis

**Affiliations:** 1 Biomedical Informatics and eHealth Laboratory, Department of Electrical and Computer Engineering, Hellenic Mediterranean University, Estavromenos, 71004 Heraklion, Greece; tsiknaki@hmu.gr; 2 Computational BioMedicine Laboratory, Institute of Computer Science, Foundation for Research and Technology—Hellas, Vassilika Vouton, 71110 Heraklion, Greece

**Keywords:** stress analysis, physiological signals, open datasets, multimodal, review

## Abstract

This review uniquely integrates open access dataset taxonomy with methodological trends in stress analysis, outlining the experimental framework and highlighting key gaps in reproducibility and FAIR compliance. In this context, stress induction methods, ground truth labeling approaches, open access datasets, computational advances, and current challenges and limitations are reported. A systematic review over the last decade (2014–2024) identified thirty-two open access affective datasets eligible for stress-related research, encompassing multimodal physiological signals, including electroencephalography (EEG), electrocardiography (ECG), electrodermal activity (EDA), and respiration (Resp), as well as behavioral measures, such as motion, audiovisual, and eye tracking data. Recent developments in signal analysis methods (2023–2025) highlight the predominance of multimodal fusion, advances in deep and self-supervised learning, personalized/adaptive models, and the growing adoption of explainable Artificial Intelligence, while machine learning approaches continue to hold a fundamental role. Despite these advances, several limitations and challenges remain, including heterogeneous experimental designs, sensor variability, limited demographic representation, data synchronization and labeling, and class imbalance. An effective experimental framework for stress research should integrate individual demographics and traits, reliable stressors, and high-quality physiological recordings within a well-defined and bias-controlled protocol, thereby producing reliable data to support and validate computational stress modeling. Continued progress in sensing, experimental standardization, and interpretable modeling is essential to produce reproducible, interpretable, and generalizable models of stress and emotions.

## 1. Introduction

Stress is the body’s physiological and psychological response to external or internal demands or threats, whether perceived or real, the so-called stimuli or stressor, that disrupt homeostasis. It is a natural adaptive mechanism that plays a crucial role in human survival, preparing individuals to respond effectively to challenges. Although low intensity short-term stress can act as beneficial, enhancing alertness, motivation, and performance [1], when intense or prolonged exposure to stressors occurs, it leads to chronic stress, which has crucial effects on both physical and mental health [2,3,4]. Stress is now recognized as a major global health concern, affecting well-being, productivity, and disease susceptibility [5]. The COVID-19 pandemic triggered an unprecedented rise in global stress levels, with reports showing a 25% increase in anxiety and depression prevalence worldwide [6]. A large-scale study of over 2.4 million responses from 149 countries from 2007 to 2021, published in 2024, reported a global increase in the incidence of emotional stress [7]. Alarmingly, the prevalence of anxiety is also a major concern for adolescents and young adults (born between 1997 and 2012) with anxiety rates to exceed those of the past three generations [8]. Studies highlight that chronic stress is associated with cognitive impairments, neurodegenerative disorders, cardiovascular diseases, and mental health conditions such as anxiety and depression [4,5]. The importance of accurate stress assessment has become increasingly evident, particularly in response to modern societal challenges [9]. Stress research has expanded across multiple scientific domains, including psychology, neuroscience, physiology, and computer science. Developing effective stress assessment methods enables accurate detection and intervention against the negative impacts of stress. Traditional stress measurement approaches, such as self-report questionnaires, are widely used but often limited by subjective bias. Physiological signals provide a valuable and reliable insight into the body’s stress response. The activation of the stress mechanism is reflected as recognizable changes in the physiological signal’s patterns, which occur involuntarily and therefore are less prone to conscious control and subjective bias [10,11]. Consequently, researchers are turning to biometric and physiological-based assessments, leveraging affective computing, wearable sensors, and neuroscience techniques to provide objective, real-time stress monitoring [6,12,13].

**Figure 1:**
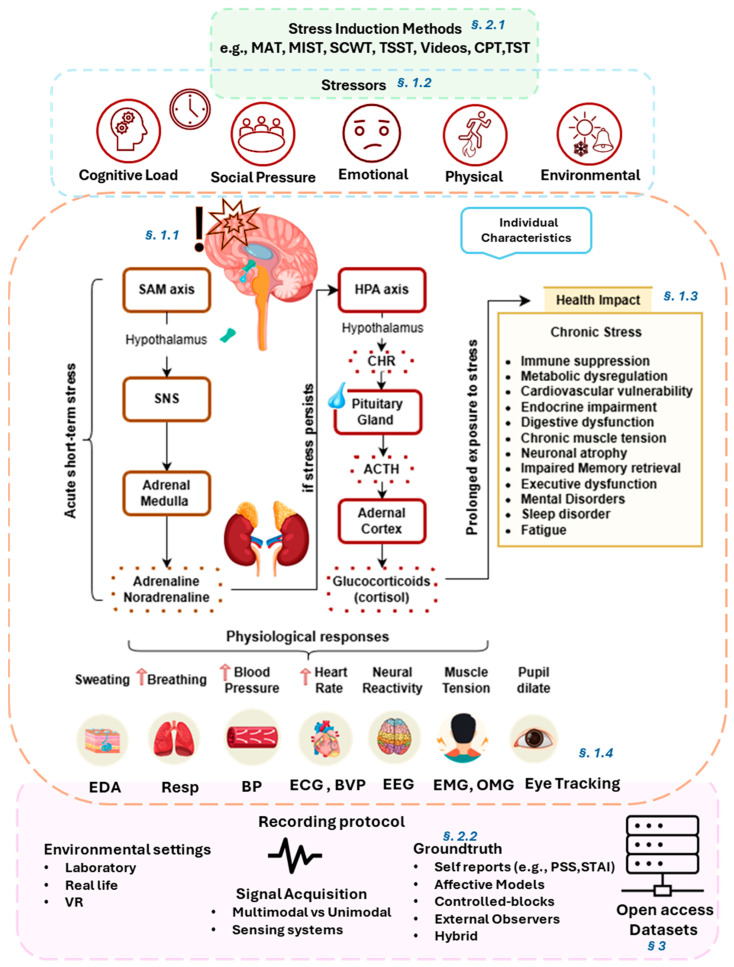
Overview of the stress response within the experimental framework for stress analysis: From stress induction methods and types of stressors to the activation of stress mechanism (SAM and HPA axis), physiological responses, health impact, and signal recording processes. Annotations of (§) refer to the corresponding sections where the details are described. The (↑) indicate increase.

The primary aim of this work is to provide an overview of the framework and methodologies followed in stress analysis based on physiological signals. This is addressed by uniquely integrating open access dataset taxonomy with key elements of the experimental framework that raise methodological challenges, identifying current trends, and highlighting reproducibility and FAIR compliance gaps. This work is structured as follows: The remaining Section 1 provides an introductory overview of the main concepts and traits of stress, by presenting its core mechanisms, types, effects, and relevant biosignals; Section 2 delves into the aspects of the experimental framework by describing its key elements and analyzing the stress induction methods and approaches for establishing ground truth; Section 3 presents a systematic review of open access affective computing datasets of the physiological signals for stress analysis according to PRISMA 2020 [14]. A schematic representation illustrating the interconnections between key elements of the experimental framework, stress mechanism, and the recording protocol leading to dataset development is presented in Figure 1, along with annotations (§) indicating the corresponding section. Following this, Section 4 outlines computational advances by presenting indicative works that reflect the current methodological trends in stress research (2022–2025); Section 5 highlights the key findings of this study, the challenges and limitations in the research domain, and addresses future research directions; Section 6 concludes the main findings.

### 1.1. Stress Mechanism

Stress is a complex and dynamic process, involving intricate interactions between the nervous, endocrine, and immune systems. When facing a stressor, the brain activates two primary physiological pathways: the sympathetic–adreno–medullary (SAM) axis and the hypothalamic–pituitary–adrenal (HPA) axis. The SAM axis is the pathway from which the brain through the hypothalamus stimulates the sympathetic system of the autonomic nervous system (ANS), which then signals the adrenal medulla to release adrenaline (epinephrine) and noradrenaline (norepinephrine), for short-term stress [15]. These activations result in physiological changes such as increased heart rate, blood pressure, respiration, and glucose mobilization, facilitating enhanced physical action, for a person to engage in stress or “fight or flight” response. However, if stress persists, the HPA axis is activated, where the hypothalamus releases corticotropin-releasing hormone (CRH), stimulating the pituitary gland to secrete adrenocorticotropic hormone (ACTH). The ACTH in turn triggers the adrenal cortex to the secretion of glucocorticoids (cortisol) [15,16]. While cortisol helps regulate metabolism, immune responses, and energy production during stress, prolonged elevation can contribute to chronic inflammation, immune suppression, and metabolic dysregulation [17]. The stress hormones cause the decreased function of neurons in the hippocampus, where new long-term memories are stored, and in the frontal lobes, where processes of attention, decision making, working memory, filtering of information, and judgment, are taking place [18,19,20].

### 1.2. Types of Stress

Defining the types of stress is not a straightforward process due to its nonlinear and multifaceted nature. An approach to discriminate stress in different types is based on the stressor’s source type and its dimensions, namely, duration, intensity controllability, and predictability [21,22]. Based on the duration of experiencing a stressful event/situation, stress can be described as (a) acute, where short-term stress is triggered by immediate stressors or challenges; (b) episodic acute, where individuals face frequent occurrences of acute stress; and (c) chronic stress, where individuals experience long-term exposure to stressful situations [23]. In another point of view, stress can be described based on the source of where stress originates from, as (a) physical, which focuses on the body’s response and could stem from body strain/illness, fatigue, sleep deprivation, chronic pain, etc.; (b) psychological, where individuals’ emotions and cognitive processes are disturbed, resulting in intense feelings such as sadness, fear, anxiety, overthinking, worry, and others; (c) environmental, which originates from external surroundings, such as noise, pollution, extreme weather conditions, discomfort, or unsafe conditions [23]. As these are broad categories, further analysis could follow based on major factors that occupy society, such as workplace stress, that emerges from professional demands and pressures, and social stress, that arises from interpersonal and societal factors, often manifesting through peer pressure, social isolation, or cyberbullying. Taking into consideration the intensity and the complexity and/or predictability of the stressor, another important type of stress is the traumatic stress which results from the exposure of the individual to traumatic events such as natural disasters, acts of violence, accidents, and the death of a loved one [24]. Long-lasting and overwhelming symptoms of traumatic stress can lead to post-traumatic stress disorder (PTSD), which may result, among others, in intrusive memories, avoidance behaviors, and hyperarousal. An ambiguous approach [25] that is also met in the literature is the classification of stress to positive “eustress” as enhancing well-being, and to negative “distress” which harms health. However, this approach oversimplifies the complex network of stress based on the outcome of the situation rather than the effects in the spectrum of stress responses [18,26,27].

### 1.3. Physiological and Psychological Effects

Recognizing the effects of stress in health, the World Health Organization (WHO) in the 11th reversion of the International Classification of Diseases (ICD) [28] incorporated a new class of mental disorders specifically associated with stress, including PTSD (6B40) and adjustment disorder (6B43), among others. Meanwhile, in the section of “Factors influencing health status or contact with health services”, burn-out (QD85) was introduced as a syndrome resulting from chronic workplace stress that has not been successfully managed, and acute stress reaction (QE84) was moved, as a normal reaction that is expected to be resolved within a short period of time [28,29]. Several stress management techniques have been proposed to help individuals cope with and adapt to life stressors, promoting physiological recovery and resilience, described as the ability to recover from adversity and maintain a high utility state [30,31]. Following the principles of Mind–Body Medicine [32,33], the BERN framework [34,35] was proposed suggesting adjustments in Behavior (social interaction and support, pleasurable activities, pacing, cognitive behavioral therapy (CBT), motivational and positive psychology), Exercise (an/aerobic physical activity), Relaxation techniques (mediation, mindfulness, spirituality, sleep hygiene), and Nutrition (balanced diet). These interventions act on the physiological regulatory reward and motivation circuitries in the CNS, supporting self-regulation and stress reduction. Rogerson et al. [36], through a systematic review and meta-analysis of studies from 1992 to 2022, evaluated the effectiveness of several stress management interventions on cortisol levels, grouped in four categories. These included Mindfulness and Meditation (e.g., mindfulness meditation, mindfulness-based stress reduction (MBSR), and mindfulness-based cognitive therapy (MBCT), Relaxation (e.g., muscle and breathing relaxation exercises), Mind–Body Training (e.g., yoga and biofeedback), and Talking Therapies (e.g., psychological interventions involving one-on-one or group, such as CBT). Among the four groups of intervention, Mindfulness and Meditation and Relaxation techniques were most effective. In addition to self-regulatory and behavioral approaches, pharmacological approaches are also studied for the effect of anxiolytic medications (i.e., benzodiazepines) and antidepressants on modulating stress response on acute stress [37]. Nevertheless, when stress is neither accurately assessed nor effectively managed, or when stressors are prolonged and excessive, the body’s stress response becomes chronically activated, leading to a cascade of adverse physiological and psychological effects.

Physiologically, chronic stress can lead to significant disruptions in multiple systems. The cardiovascular system is particularly vulnerable, as prolonged stress increases the risk of hypertension, atherosclerosis, heart attacks, and strokes due to sustained elevations in blood pressure and heart rate [38]. The endocrine system is also affected, with chronic cortisol release leading to insulin resistance, weight gain, and an increased risk of Type 2 diabetes [39]. Stress suppresses the immune system, making individuals more susceptible to infections and increasing inflammation, which is linked to autoimmune diseases and chronic conditions such as arthritis [17]. The digestive system experiences dysfunction as well, with stress contributing to acid reflux, irritable bowel syndrome, nausea, and appetite changes [40]. Furthermore, chronic muscle tension from prolonged stress can lead to migraines, headaches, and musculoskeletal pain [41].

Psychologically, stress has profound effects on cognition, emotion, and behavior, largely due to its impact on key brain regions, such as the hippocampus, prefrontal cortex, amygdala, and hypothalamus [42]. Chronic stress not only affects the activity of these brain regions, but it can even cause structural changes with long-lasting effects [43]. The hippocampus, responsible for memory and learning, is particularly sensitive to chronic stress, as prolonged cortisol exposure can lead to neuronal atrophy and impaired memory retrieval [43]. The prefrontal cortex (PFC), which governs decision making, impulse control, and executive function, experiences reduced activity under stress, leading to poor problem-solving skills, heightened impulsivity, and difficulty focusing [19,20,43]. Meanwhile, the amygdala, part of the limbic system at the end of the hippocampus, which is the brain’s fear processing center, becomes hyperactive, resulting in increased anxiety, emotional instability, and hypervigilance [24]. This imbalance contributes to mood disorders such as depression, generalized anxiety disorder (GAD), and PTSD. Both acute and chronic stress cause dendritic growth in neuros in the amygdala, which result in increased levels of anxiety and aggression [19,44]. Stress also disrupts sleep cycles, leading to insomnia, restless sleep, and fatigue, further exacerbating emotional dysregulation and cognitive impairment [45].

The effects of stress that an individual will experience vary based on the stressor’s type and characteristics, how it will be perceived, and on the combination of individuals’ psychological, biological, and developmental factors, including age and sex. The stressor and therefore the type of stress being stimulated alter differently the degree of activation of the stress mechanism, i.e., the sympathetic branch of ANS and the HPA axis, producing distinct neurophysiological patterns but also overlapping. Acute physical stress typically produces reactive responses, communicated directly to the paraventricular nucleus of the hypothalamus via monosynaptic relays from the sensory organs, resulting in the rapid activation of HPA axis [46], whereas in the case of acute psychological stress, the response is more anticipatory as it requires processing from the limbic system, including the amygdala and hippocampus, to assess a threat [46]. These activations can be captured as increased cortisol secretion, increased heart rate, reduced heart rate variability, elevated electrodermal activity, faster and irregular respiration, toned muscle tension, cortical changes as of reduced alpha (relaxation state), and increased beta (cognitive processes and alertness) brain waves power, which typically would appear more rapid, intense, and short-termed in physical stress, and relatively more sustained in psychological stress with enhanced cortical activity [10,42,43,44,45]. However, the overall trajectory of the HPA axis response and subsequently the reflection on the physiological signals is majorly affected by the stressor intensity and duration [46], among other factors. In contrast, in cases of chronic stress, sustained activation of the HPA axis is observed, which manifests as sensitized reactions or in extreme cases hyporesponsiveness in new stressors [46], which is captured in physiological signals as a baseline shift and long-term dysregulation [10].

Evidence shows that both sex and age influence the stress response. Sex differences are linked with gonadal steroids, testosterone in males, and estradiol (E2) and progesterone in females, with additional variability appearing in menstrual cycle phase (lower level of cortisol and limbic activation during high estrogen state) [1,47,48]. Gonadal steroids modulate the function of the HPA axis with estradiol to enhance reactivity while testosterone weakens reactivity to stress [1,47,48]. Age affects stress responses by altering the sensitivity and regulation of the HPA axis over time, in respect to gonadal steroid developments and sex. Typically, adolescents exhibit stronger and prolonged responses with less efficient and slower recovery, adults show more balanced and adaptive mechanisms with faster recovery, and older adults present weaker acute responses and slow recovery [49]. However, most biomedical studies on humans deal with young adults (18–35) [3], and older adults (above 65) [50]. The underrepresented age group of 35–65 deals with important changes in the HPA axis and gonadal steroids, with testosterone in males to naturally decline and post-menopausal females to face important declines in estradiol and progesterone, which are related to stress response and related disorders, such as depression and anxiety [48]. Research highlights the need for more studies to investigate the sex differences, elaborating age groups, and gonadal steroids state (menstrual cycle phase and menopausal in female, testosterone decline in male), with caution on cortisol analysis, stressor characteristics, and psychological attributes, as well as other underlying mechanisms [1,47,48,49,50].

It is important to highlight that there are no specific response patterns, nor identical responses of individuals to a stressor [51,52]; nonetheless, stress consistently impacts physiological and psychological systems leading to recognizable associated effects. The variability in responses stems from individual differences, the nature of the stressor itself, including intensity and duration, as well as how individuals perceive the stressor’s controllability and predictability [49,52,53]. Factors such as age, sex, genetics, prior stressor exposure, coping mechanisms, perceived stress, early life experiences, personality traits, culture, psychological state, and physical health further contribute to individual differences in stress responses [49,54,55]. These factors interact in complex ways to shape individual stress responses.

### 1.4. Biosignals in Stress Analysis

In stress analysis, biosignals provide objective and quantifiable markers of stress-related physiological changes across the autonomic, cardiovascular, peripheral, and central nervous systems. Various signals such as ECG, photoplethysmography (PPG), EEG, EDA, Resp, skin temperature (ST), and electromyography (EMG) have been proven to reflect the physiological changes in stress response [11]. Each signal provides a unique window into the stress response, and, when combined, they allow for a comprehensive investigation of how people respond to various stressors [56], while also allowing a more distinct view of the physiological responses under overlapping mechanisms. The selection of biosignals depends on the type of stress being studied (e.g., cognitive, emotional, physical), the research objectives, and the experimental design’s structure and limits.

One of the most widely utilized biosignals is ECG, which measures the electrical activity of the heart. From ECG, the heart rate (HR) and heart rate variability (HRV), which is the variation in the time distance between successive heartbeats (RR intervals), are derived as key indicators of stress. ECG is directly measured from the chest using sensors such as chest-strap electrodes (e.g., Zephyr BioHarness [57]) and electrode patches (e.g., The Shimmer3R ECG [58]). HR and HRV are highly associated in both physiological and mathematical means [59]. An increase in HR causes a decrease in HRV and vice versa [59]. Under stress an increase in HR is linked with higher sympathetic nervous system activity [11,60]. HRV is considered an indicator of autonomic balance reflecting the interaction between sympathetic and parasympathetic systems [59,61,62]. Reduced HRV is linked with reduced parasympathetic vagal activity [61,63], while stress is particularly associated with low parasympathetic activity, indicated by the decrease in the high-frequency (HF) band and an increase in the low-frequency (LF) band of HRV [62].

PPG is an optical method used to detect changes in blood volume in the microvascular layer of tissue, by measuring variations in light absorption and reflection during heartbeat. A PPG sensor consists of a light source, typically infrared or a green LED, and a photodetector (photodiode). The light source emits light to the skin, which partially is absorbed through biological tissues and partially is reflected. The photodetector senses the reflected light, which is converted into an electrical signal proportional to its intensity [64,65]. The obtained PPG signal consists of a pulsatile waveform alternating current (AC) component and a slow varying baseline direct current (DC) component [66]. The AC component of the PPG signal represents the blood volume pulse (BVP), which refers to the pulsatile blood volume changes [66,67]. From the BVP signals, the pulse rate (PR) and pulse rate variability (PRV) are estimated, which are considered valid substitutes of the HR and HRV of ECG, and therefore interpretations of their changes in relation to stress are similar [68]. Moreover, respiratory-induced variations, BVP blood oxygen saturation level (SpO2), and blood pressure (BP) can also be estimated [11,67,68]. PPG sensors are extensively used in stress research, as they allow continuous monitoring through low-cost and easily wearable solutions such as wristbands and smartwatches, with the Empatica E4 wristband [69] to be the most utilized sensor.

EEG reflects the electrical activity of the brain and is especially useful for in-depth analysis of cognitive and emotional stress [70]. The EEG signal is analyzed to each frequency band/rhythm, namely, alpha (8–13 Hz), beta (13–30 Hz), gamma (30–70 Hz), delta (0.5–4 Hz), and theta (4–8 Hz). Stress evokes characteristic changes in EEG frequency bands, including frontal alpha asymmetry, reduced alpha power (emotional arousal), altered beta activity in the temporal and parietal lobes, primarily occurring in the brain’s left anterior origin (mental engagement, stress, or anxiety), and increased frontal delta and theta (cognitive load, challenges) wave power [71,72,73,74,75]. In affective computing research, various sensing systems, mainly wireless and portable, are used, with wet or dry electrodes, such as the Muse [76], NeuroSky [77], Emotiv [78], BioSemi [79], and g.tec [80] EEG headsets.

EDA, also referred to as galvanic skin response (GSR), captures the electrical conductance of the skin. Under stress, the sweat gland activity promotes an increase in EDA, which corresponds to a direct output of sympathetic nervous system activation [56]. EDA is highly sensitive to emotional stress, especially during tasks involving fear, anxiety, or social stress (i.e., TSST, Cyberball) and startle responses. Typically, it is measured using finger or palm electrodes (i.e., Biopac [81], Shimmer [58]) or via wearable sensors (i.e., Empatica E4 [69]).

Respiration is another biosignal of study, as stress often disrupts normal breathing patterns, with breathing becoming faster and shallower, particularly in conditions of psychological strain [11]. To identify stress-induced breathing irregularities, respiratory rate and rhythm are measured using chest belts or impedance sensors. Other peripheral measures that serve as indicators of stress include skin temperature, which decreases due to vasoconstriction, and electromyography, which measures muscle tension typically in the forehead or shoulders, indicating somatic stress or physical strain [11]. These signals are monitored through thermistors and surface EMG sensors, respectively. A non-contact technique to capture muscle activity was introduced in 2015 by Muhammed et al. [82], namely, optomyography (OMG), as an alternative to EMG. OMG utilizes infrared optical sensors to measure muscle activity based on the optical reflectance changes in the skin surface when muscles contract and deform [82,83]. For the analysis of facial expressions through facial muscle activity based on OMG, the OCOsense™ smart glasses are used [84,85].

Biosignals can provide a robust framework for stress assessment incorporating a multimodal approach; however, the trustworthiness of sensing these signals comes with challenges and limitations. Limited to the sensing capabilities of each device, further challenges include motion artifacts, environmental noise, variability in sensor placement and contact, reliability of ground truth, processing techniques and signal fidelity, data synchronization, and labeling, as well as the model’s accuracy, scalability, and generalizability [86,87,88,89].

## 2. Experimental Framework for Stress Analysis

Towards the development of reliable and accurate detection models, key elements of the experimental framework, including the design and methodological decisions of data collection process that could influence validity and may induce bias, must be considered. The environmental setting, laboratory or real-life, determines the degree of manipulation of the independent variable (i.e., stress or affect induction method) and the level of control of extraneous variables (e.g., environmental noise, movement artifacts, sensor displacement, distraction, temperature, time of day) or confound variables (e.g., mood, exercise, smoking, other concurrent stimuli) [90]. Laboratory environments allow precise manipulation and control of the experimental variables, while real-world settings introduce realistic scenarios of application with the cost of variability and noise.

The sample size is another important factor, as an insufficient number of participants may fail to demonstrate the desired effect or to estimate the frequency of occurrence [91]. Several approaches exist for determining the appropriate sample size based on the study design approach, statistical analysis, effect size, confidence levels, and regression analysis, with available software tools also for estimation [92]. A recent study [93] on the sample size for neurophysiological data analysis reported that for determining the minimum number of participants, the task length should also be taken into consideration, resulting in the decision that for the investigated case study a size of 24 participants for 30 s tasks was acceptable. The inclusion of balanced age groups, sex, and sociocultural profiles of participants is also important as these factors modulate the stress responses and contribute to inter-individual variability.

Depending on the study objectives, the selection of physiological and behavioral measurements (EEG, ECG, EDA, EMG, BVP, ST, Resp, facial video, eye tracking, etc.) and the corresponding sensing devices requires careful consideration of each signal’s unique characteristics and specifications [94], while supporting multidimensional emotion analysis requires a multimodal approach with EEG to play a key role [95,96,97]. In multimodal approaches, data synchronization is also needed to ensure temporal alignment across devices and stimuli.

Similarly, the structure of the experimental protocol is critical, which is typically organized as successive blocks of different affective states with intermediate baseline periods, or incorporating relaxation or meditation blocks to restore physiological balance after inducing an intense emotion [98]. When involving multiple affective conditions, varying the order of affective blocks can reduce potential bias from participant familiarization or expectancy effects [99]. Another key parameter that can be managed by the experimental structure is the balance of affective states, by equal time representation. However, ethical concerns arise when inducing negative emotional states for prolonged periods, while there is also evidence that people tend to maintain positive emotional states better than they do negative states [100].

Two core elements of the experimental framework are how stress is experimentally induced (independent variable) and how the ground truth of affective states is considered. As these methodological approaches critically influence the integrity and validity of the approach, the most widely used stress induction methods and ground truth approaches based on the procedures followed in the publications included in this work, but which are not limited to these, are reported in more detail in Section 2.1 and Section 2.2.

### 2.1. Stress Induction Methods

Stress response is elicited by any external or internal stimulus that disrupts an individual’s physiological and/or psychological balance, the so-called stressor. Stressors, relative to types of stress described in Section 2.1, can be broadly categorized into (a) physical stressors, such as physical strain, pain, sensory overload, and environmental disturbances like exposure to loud noise or intense light; and (b) psychological stressors, including cognitive overload, emotional challenge, and social evaluation [101]. The dimensions of the duration, intensity, predictability, and controllability of the stressor play a crucial role in shaping the body’s physiological responses [21,22]. Duration differentiates between acute stressors, which cause short-lived responses, and chronic stressors, which persist over time, leading to long-term consequences [53]. Intensity influences the severity of autonomic activation, with high-intensity stressors provoking stronger changes. Predictability and controllability affect how individuals anticipate and adapt to stress, emphasizing the cognitive and perceptual aspects of stress [21], with unpredictable stressors often inducing greater physiological arousal compared to controlled, expected stimuli. Furthermore, the complexity of a stressor relatively imposes increased cognitive, sensory, or physical load and effort, influencing task performance, attention, and emotional processing. These factors impact the reliability of physiological signals recorded in laboratory settings using stress induction methods for the development of computational models. Table 1 summarizes the various stress induction methods used in laboratory settings, detailing their descriptions and corresponding stress-related types, with relevant citations on the original study or/and major updates.

**Table 1:** Overview of stress induction methods.

Ref.	Stress Induction Method	Description	Related Stress Type
[102]	Mental Arithmetic Task (MAT)	Solving arithmetic problems under time constraints. Stress increases with difficulty and pressure.	Cognitive Load and Social (Task Performance)
[103]	Montreal Imaging Stress Task (MIST)	Mental arithmetic task with random failure feedback, even when correct, inducing frustration.	Cognitive Load and Social (Task Performance)
[104]	Paced Auditory Serial Addition Test (PASAT)	Listening to numbers and continuously summing the last two heard while time constraints increase.	Cognitive Load and Social (Task Performance)
[105,106]	Stroop Color Word Test (SCWT)	Naming the color of an incongruent word (e.g., “BLUE” written in red). Requires inhibitory control and attention.	Cognitive Load and Task Performance
[107]	Multitasking Challenge	Subjects are required to perform multiple simultaneous tasks to induce cognitive overload.	Cognitive Load and Social (Task Performance)
[108,109]	Multi-Attribute Task Battery -II	A computer-based set of tasks designed to evaluate simultaneous performance of monitoring, dynamic resource management, and tracking tasks (aircraft crewmembers, with freedom to use by non-pilot subjects).	Cognitive Load and Task Performance
[110,111]	Time Pressure Tasks	Participants must complete cognitive or motor tasks under strict time constraints.	Cognitive Load and Task Performance
[112]	Reading Span Task (RSPAN)	Memory span task exploring working memory, cognitive processing, and reading comprehension.	Memory, Cognitive Load, and Task Performance
[113,114]	Trier Social Stress Test (TSST)	5 min of public speech, and 5 min of mental arithmetic task in front of a panel of evaluators (two to five) for 15 min.	Social and Cognitive Load (Task Performance)
[115]	Maastricht Acute Stress Test (MAST)	Combination of the Trier Social Stress Test and the Cold Pressor Test.	Social and Cognitive Load, Environmental and Physical
[116,117]	International Affective Digitized Sounds (IADS) and (IADS-E)	Listening to distressing sounds (screams, alarms) to evoke stress.	Acoustic and Emotional
[118]	International Affective Picture System (IAPS)	Exposure to emotionally charged images (negative, neutral, positive).	Emotional
[119]	Cyberball Social Exclusion Task	A virtual game in which participants are intentionally excluded, inducing social rejection stress.	Social
[120]	Public Speaking Task	Impromptu speech task with evaluation from peers or judges.	Social
[121]	Memory Recall of Traumatic Events	Participants recall past traumatic events, activating stress responses.	PTSD and Emotional
[122]	Cold Pressor Test (CPT)	Subjects immerse their hand in ice-cold water (0–4 °C) to induce a physiological stress response.	Environmental and Physical
[123]	Thermal Stress Test (TST)	Exposure to extreme heat or cold temperatures tests thermoregulation under stress.	Environmental and Physical
[124]	Exposure to Light (Photostimulation)	Sudden exposure to bright or flickering lights induces sensory processing stress.	Environmental and Physical (Visual Sensory Overload)
[125,126]	Hyperventilation Challenge	Subjects are asked to breathe rapidly to mimic anxiety-like symptoms and autonomic dysfunction.	Physical
[127]	Exposure to Noise	Subjects are exposed to loud, unpredictable noises such as alarms, construction sounds, or white noise.	Environmental and Physical (Acoustic Sensory Overload)

### 2.2. Ground Truth of Affective States

The ground truth of affective states refers to the actual emotional state of the individual during an experimental protocol, which researchers use to develop physiological signals datasets that serve as input for computational stress analysis models. The reliability of ground truth is critical for the accurate training, validation, and evaluation of the computational approaches and subsequently their comparison. Table 2 presents the standardized affective assessment questionnaires that are used in affective computing research and particularly those which deal with stress-relevant dimensions, alongside a short description, the affective condition relevant to stress, and the citation of the original study and/or major updates.

**Table 2:** Standardized affective assessment questionnaires.

Ref.	Questionnaire	Description	Affect Condition
[128,129]	Perceived Stress Scale (PSS) and (PSS-10)	Measures perceived stress.	General Perceived Stress
[130,131]	Self-Assessment Manikin (SAM)	A visual scale for valence (emotion), arousal (activation), and dominance (control).	Positive/Negative Emotion
[132]	Positive and Negative Affect Schedule (PANAS)	Measures positive and negative emotional states separately to infer affective stress responses (Positive/Negative Affect).	Positive/Negative Emotion
[133]	State-Trait Anxiety Inventory (STAI)	Differentiates temporary (state) vs. long-term (trait) anxiety.	Anxiety (acute and chronic stress)
[134]	Depression Anxiety Stress Scales (DASS-21)	Evaluates the negative emotional states of depression, anxiety, and stress.	Anxiety, Depression, Stress
[135]	Beck Anxiety Inventory (BAI)	Assesses physical symptoms of anxiety.	Anxiety and PTSD
[136]	Hamilton Anxiety Rating Scale (HAM-A)	Evaluates clinical anxiety severity.	Anxiety and PTSD
[137,138]	Post Trauma Cognitions Inventory (PTCI) and (PTCI-9)	Evaluates negative trauma-related thoughts and beliefs.	PTSD
[139]	Post-traumatic Stress Disorder Checklist for DSM-5 (PCL-5)	Measures PTSD symptoms in line with DSM-5 diagnostic criteria.	PTSD
[140]	General Health Questionnaire-12 (GHQ-12)	Assesses mental health problems, specifically psychological distress such as anxiety, depression, and social dysfunction.	General mental and health psychological distress
[141]	Stress Response Inventory (SRI)	Assesses emotional, cognitive, somatic, and behavioral stress responses.	Stress response
[142]	Attentional Control Scale	Assesses an individual’s capacity for attentional control, including focusing attention, shifting attention, and flexibly controlling thoughts.	Cognitive and Attentional Control Under Stress
[143]	NASA Task Load Index (NASA-TLX)	Assesses cognitive workload in tasks under pressure.	Cognitive and Task-Related Stress
[144,145]	Rating Scale Mental Effort (RSME)	Measures subjective mental workload and effort during tasks.	Cognitive
[146]	Daily Stress Inventory (DSI)	Captures frequency and intensity of daily stressful events.	Daily Hassles and Minor Stressors
[147]	Cambridge Cognitive Assessment -Revised (CAMCOG-R)	Assesses cognitive function including memory, orientation, and attention.	Cognitive Function and Stress-Related Decline
[148]	Mini-Mental State Examination (MMSE)	Screen cognitive function, often used to rule out cognitive decline.	Cognitive Function and Mental Impairment
[149]	Cohen–Hoberman Inventory of Physical Symptoms (CHIPS)	Assesses physical symptoms commonly associated with stress.	Physical symptoms
[150]	Stress Mindset Measure (SMM)	Assesses beliefs about the nature and the effects of stress (positive or negative).	Personality Traits: Stress Beliefs and Mindset Influence
[151]	Eysenck Personality Questionnaire (EPQ)	A brief measure of three broad personality dimensions: Psychoticism, Extraversion, and Neuroticism.	Personality Traits: Stress susceptibility
[152]	Big Five Inventory 10 Item Scale (BFI-10)	A brief measure of five broad personality dimensions: Openness, Conscientiousness, Extraversion, Agreeableness, and Neuroticism.	Personality Traits: Stress susceptibility

To establish ground truth, subjective, controlled, observable, and combinations of those approaches can be used. The subjective approach refers to the use of standardized scales where individuals self-report their emotional state/condition before, during, and/or after the experimental protocol. The subjective report is of high importance as the feeling of the individual is the essence of an emotion [153]; however, it can induce bias, including, among others, the introspective ability of the individuals to assess themselves and the truth of the answer versus the social acceptability of the answers. In the controlled approach researchers use, mainly in laboratory settings, emotion induction methods (as described in Section 2.1 for stress) that have been validated, relying on the assumption that the inquest emotion has been effectively elicited within a task-relevant time frame. This approach may introduce bias by presuming consistent emotional responses across individuals, potentially overlooking personal differences in how emotions are experienced or expressed, i.e., a video labeled as funny may evoke amusement in one person but appear neutral to another. Conversely, in the observable approach, one or more researchers and/or clinical professionals monitor the experimental process and annotate the recorded physiological signals using event markers based on the interpretation of the individual’s emotional reactions, which subsequently is limited to the objective judgment of the observer.

One of the most widely adopted approaches for mapping the affective state of individuals and evaluating the integrity of the stimuli in experimental protocols of psychophysiological research is Russell’s Circumplex Model of Affect [154]. According to Russell’s Circumplex Model, emotions can be described within a two-dimensional space defined by Valence (pleasant–unpleasant) and Arousal (low–high), producing four quadrants that reflect distinct emotional states. The High-Arousal/Positive-Valence quadrant reflects energizing pleasant states such as excitement and joy, typically accompanied by sympathetic activation perceived as beneficial. The Low-Arousal/Positive-Valence quadrant captures calm and pleasant states, including relaxation and serenity, where parasympathetic dominance and reduced physiological activation prevail. In contrast, the High-Arousal/Negative-Valence quadrant represents unpleasant and intense states such as fear, anger, and stress, which are strongly linked to sympathetic hyperactivation and therefore increased physiological activation. Finally, the Low-Arousal/Negative-Valence quadrant describes unpleasant states, including sadness, depression, and fatigue. Relatively, the Three-Dimensional Emotional Space Model [155], also known as PAD, adds the Dominance dimension which reflects the feeling of control or power over a situation or emotion. Subjective reporting by participants is usually performed through tools such as the Self-Assessment Manikin (SAM), a non-verbal pictorial assessment technique that directly measures the pleasure, arousal, and dominance of a person’s response to a stimulus [131].

As emotions are complex, multidimensional, and influenced by subjective, behavioral, and physiological factors, a combined approach that integrates these dimensions along with recordings of physiological signals could be the best practice to establish a reliable ground truth.

## 3. Open Datasets

**Figure 2:**
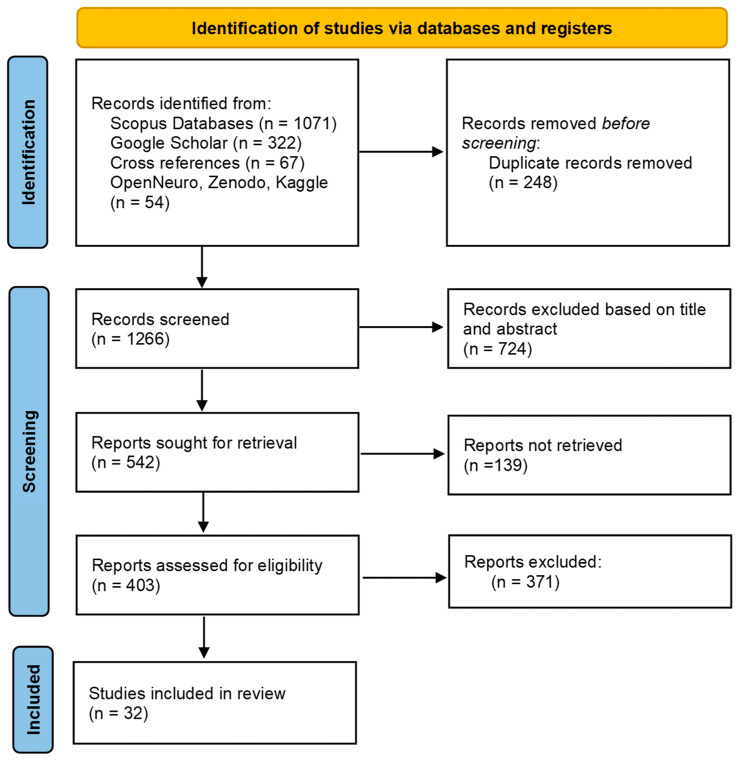
The PRISMA flow diagram that maps the phases of the systematic review conducted for open datasets suitable for stress analysis based on physiological signals.

Over the last decade the growing number of physiological stress and emotion datasets reflects the research focus for in-depth analysis of the underlying neurophysiological mechanisms, but also the evolution of signal processing and modeling methods. Open access datasets play a fundamental role in advancing research, enabling benchmarking, reproducibility, and the development of advanced multimodal and crossmodal models. Accordingly, we conducted a systematic review, following the Preferred Reporting Items for Systematic Reviews and Meta-Analyses (PRISMA 2020) guidelines [14], on open access affective computing datasets eligible for stress analysis based on physiological signals, published from 2014 to 2024, addressing a gap in the current literature. A PRISMA flow diagram is presented in Figure 2, summarizing the results of the identification, screening, eligibility, and inclusion phases of the methodological process.

Search Strategy

A structured search strategy was performed in two periods between September 2024 to November 2024 and the first six months of 2025, across the electronic academic databases of Scopus and Google Scholar and the online research repositories OpenNeuro, Zenodo, and Kaggle, while further cross reference works were exploited. The following search query was used for Scopus and Google Scholar: (“dataset” OR “database”) AND (“stress” OR “anxiety” OR “affect” OR “emotion”) AND (“biosignals” OR “physiological signals” OR “EEG” OR “ECG” OR “PPG” OR “EDA” OR “GSR”). For the search on OpenNeuro, the modality of EEG was chosen and combined with the keywords describing the affective condition. For the search on Zenodo, (type: dataset) and the rest of the query used in academic datasets for the affective condition and biosignals was used. For the search on Kaggle, the Dataset section was launched and combined with the keywords describing the affective condition and/or the biosignals. The searches were limited to the years between 2014 and 2024. Search results were included in the Mendeley Reference Manager and duplicates were excluded. Initiating the search in the Scopus database, as one of the largest databases, and resulting in a large number of studies, we did not expand the search into databases such as PubMed, IEEE Xplore, and ACM Digital Library, which is a limitation of this review. However, due to the exploitation on Google Scholar, the online repositories, and cross references, we consider that the majority of open datasets has been identified. Another limitation is that a protocol was not pre-registered for this systematic review.

Inclusion and exclusion criteria

Studies describing open access datasets were included if the following criteria were met:

- Accessibility: Sufficient and clear description of the terms and conditions of dataset availability and accessibility for research and educational purposes.
- Physiological signals: Combination of at least two signal modalities (EEG, EDA/GSR, ECG/BVP/PPG, EMG/OMG, Resp), or single in the case of EEG.
- Experimental framework: Documentation of sufficient details of the experimental protocol, including data acquisition, stimulus/elicitation method, ground truth labeling, and baseline.
- Eligibility for stress analysis.

Studies were considered eligible for stress analysis if at least one of the following criteria were met:

- Scientific relevance: Explicit focus on stress by experimental protocol design.
- Affective model: Comply to Russell’s Circumplex Model of Affect or the PAD framework for identifying valence/arousal states.
- Stimuli: Use of stimuli validated in stress-related studies.
- Ground truth: Inclusion of stress-relevant assessment questionnaires.

Studies involving minors, participants facing health conditions, or only static images as stimuli were excluded.

Selection Process

After the initial identification and removal of duplicates, studies were screened based on the title and abstract and excluded if irrelevance or inconstancy with the objective were found. A total of 403 studies were screened with full text evaluation for meeting the inclusion criteria or excluded if exclusion criteria were met. The selection and evaluation process was performed by a single reviewer. Therefore, a limitation related to subjective judgment and potential bias in the interpretation of the predefined criteria must be acknowledged. In the final evaluation process, some studies that were initially considered eligible were excluded, after careful consideration, as not meeting the criteria explicitly. For example, the SWEET dataset [156], which incorporates physiological signals from 240 participants in their work environment, was excluded due to doubts on the terms and conditions of availability, as in the corresponding study the dataset is stated as not publicly available, but on request the data that support the study could be available, with a parallel online search to reveal no registered information online. Another example of exclusion is the MODMA dataset [157] as it includes EEG recordings from clinically diagnosed depressed patients, although it also includes healthy participants recordings. A well-structured dataset that was excluded due to the use of only pictures for stimuli is the LEMON Dataset [158], which includes magnetic resonance imaging (MRI), EEG, peripheral physiological measurements, and various behavioral data.

Results

The review revealed thirty-two open access datasets eligible for stress analysis. The results are organized in Table 3, providing an overview of those datasets, in chronological order, that could be explored in stress and affective computing research, highlighting the physiological and other signals exploited and details of the experimental protocols, i.e., the sensing equipment and the stimulus used, the affective condition studied, the assessment questionnaires used, and the demographics of participants (Number of Participants, Female/Male, Mean Age or Age Span). Although the review focuses on the years 2014–2024, two earlier releases, the DEAP and the MANHOB-HCI datasets, are also included for completeness as they have become benchmarks in affective computing research.

Among the thirty-two datasets, eleven datasets incorporate stress as a key research objective of their experimental design, namely, SWELL-KW, WESAD, IDEA, PASS, Anxiety, NURSE, MMSD, SAM 40, XR4DRAMA Stress, StressID, and WorkStress3D. The remaining twenty-one enable the indirect inference of stress by involving in the experimental design a validated stress induction stimulus, such as TSST, MAST, SCWT, MAT, or other physical or social stressors; and/or the dimensions of emotion valence and arousal; and/or the use of standardized questionnaires relevant to stress, positive/negative emotions, and valence/arousal dimensions, such as PSS, PANAS, STAI, and SAM. The datasets differ in several dimensions of the experimental design, including environmental settings, multimodal and unimodal approaches and sensing devices, types of stimuli used, ground truth, and labeling, reflecting the wide range of experimental frameworks and methodological approaches applied in affective and stress research. Beyond these dimensions, other important factors that influence benchmarking and reproducibility towards the development of advanced models include data synchronization methods, participants’ demographics, class imbalance of affective states, and the adherence to accessibility and FAIR data standards.

**Table 3:** Overview of open datasets for affective and stress-related research.

Ref.	Year	Dataset Name	Signal Modalities	Sensors/Equipment	Stimulus	Affective Condition	Questionnaires	No. Par. F/M, Age
[98]	2011	DEAP	EEG, GSR, ECG, BVP, EMG, EOG, Resp, ST, facial video	Biosemi Active II-32 active AgCl electrodes, peripheral sensors	40 one-minute emotional music video clips	Valence, arousal, dominance, liking	SAM (valence, arousal, dominance, liking, familiarity)	32, 16/16
[159]	2012	MAHNOB-HCI	EEG, ECG, EDA, Resp, ST, eye gaze, facial video	Biosemi Active II-32 active AgCl electrodes, Tobii X120, AKG mics, multi-camera video	20 emotional video clips, 28 images, implicit tagging trials	Valence, arousal, dominance, predictability, emotional labels of neutral, anxiety, amusement, sadness, joy, disgust, anger, surprise, and fear	SAM (valence, arousal, dominance), emotional keywords	27, 17/13, 26
[160]	2014	SWELL-KW	ECG, EDA, facial video, posture (Kinect), mouse/keyboard activity	TMSI Mobi, Kinect 3D, iDS FaceCam, Computer logging (uLog)	Office tasks with time pressure and email interruptions	Stress, valence, arousal, dominance, task load	NASA-TLX, SAM (valence, arousal, dominance), RSME, 1–10 scale (stress), internal control index	25, 8/17, 25
[161]	2015	SEED	EEG	ESI NeuroScan 62-electrode cap, 1000 Hz	15 film clips (5/positive, neutral, negative)	Positive, neutral, negative	Self-assessment, EPQ	15, 7/8, 23
[162]	2018	DREAMER	EEG, ECG	Emotiv EPOC, Shimmer	18 emotional video clips from films	Valence, arousal, dominance	SAM	23, 14/9, 26
[99]	2018	WESAD	ECG, EDA, EMG, Resp, ST, BVP, ACC	RespiBAN Professional (chest), Empatica E4 (wrist)	TSST, amusing videos, guided meditation	Neutral, stress, amusement	PANAS, STAI, SAM, SSSQ	15, 3/12, 27
[163]	2018	STEW	EEG	Emotiv EPOC (14 channels, 128 Hz)	SIMKAP multitasking test (visual and auditory tasks)	Mental workload (low, moderate, high)	9-point workload rating scale	48, 0/48, 22–30
[164]	2019	CLAS	ECG, PPG, EDA, ACC	Shimmer3 EDA and ECG units	MAT, logic, SCWT, DEAP video, IAPS images	Arousal, valence, concentration, cognitive load	Self-assessment questionnaires (not defined)	62, 17/45, 23
[165]	2019	DASPS	EEG	Emotiv Epoc (14 channels)	Recall real-life anxiety inducing events (e.g., loss, trauma, financial stress)	Anxiety (four levels), arousal, valence	HAM-A (pre/post), SAM (valence, arousal)	23, 13/10, 30
[166]	2019	EEGMAT	EEG	Neurocom EEG (23 channels), 500 Hz	Serial subtraction task (4-digit–2-digit), 4 min	Cognitive load	Performance-based grouping	36, 24/12, 18
[167]	2020	EEG Emotion DB	EEG	EEG Clarity BrainTech 32+ CMEEG-01, 32 channels, 256/1024 Hz	12 emotional video clips 2.5 min each	Happy, sad, fear, neutral	After each video, participants matched their feelings with one of the listed four	44, 23/21,
[168]	2020	IDEA	EEG	RMS EEG System, 24 channels used, 256 Hz	Movie clips, songs, instrumental music, complex math tasks	Pleased, cheerful, zest, relaxed, distress, anger, restlessness, sadness	Experimental design-based	14, 6/8, 20
[169]	2020	PASS	EEG, ECG, Resp, BVP, ST, ACC,	Muse 2 4-channel EEG headset, BioHarness 3, Empatica E4 wristband	Stationary biking at three speeds while playing a clam and a survival video game	Neutral, stress	NASA-TLX, BORG	48, N/A, 26
[170]	2021	AMIGOS	EEG, ECG, EDA, RGB video, depth video	Emotiv EPOC 14 channel EEG headset, Shimmer 2R, Microsoft Kinect V1	Emotional videos (short/individual, long/group)	Valence, arousal, dominance, liking, familiarity, Nine feelings: neutral, disgust, happiness, surprise, anger, fear, and sadness	SAM (valence, arousal, dominance, liking, and familiarity), BFI, PANAS	40, 13/27, 28
[171]	2021	DEAR-MULSEMEDIA	EEG, GSR, PPG	Muse 4-channel EEG headset, Shimmer GSR and PPG, fan, heater, olfaction dispenser, haptic vest	Movie clips enhanced with cold air, hot air, olfaction, haptics	Valence, arousal	SAM (valence, arousal) 9-point	18, 9/9, 20
[172]	2021	MuSe 2021/Ulm-TSST	EDA, ECG, Resp, HR, audio and video recordings, text	N/A for physiological signals; cameras, microphones	TSST: oral presentation	Valence, arousal	External raters, valence and arousal	69, 49/20, 18–39
[173]	2021	VREED	EDA, ECG, eye tracking data	FOVE-0 VR headset with eye tracking, Biopac MP150	Immersive 360° video-based virtual environments	Valence, arousal, joy, anger, calmness, sadness, relaxation, happiness, fear, anxiousness, dizziness	SAM (valence, arousal), VAS (discrete emotions), Presence Questionnaire (immersion)	34, 17/17, 25
[174]	2022	Anxiety Dataset	ECG, Resp	Biopac MP45	Anxiety inducing vs. relaxing video clips	Anxiety (pre/post-induction)	BAI, HAM-A	19, 5/14, 26
[175]	2022	NURSE	EDA, BVP, HR, ST, ACC	Empatica E4	Nurses working in a hospital during the COVID-19 outbreak	Stress	Validation survey on COVID-19/medical stressor category	15, 15/0, 30–55
[176]	2022	Emognition	EEG, BVP, EDA, ST, HR, IBI, PPI, ACC, GYRO, facial expressions	Muse 2, Empatica E4, Samsung Galaxy Watch	Video clips in nine emotions categories	Amusement, awe, enthusiasm, liking, surprise, anger, disgust, fear, sadness, valence, arousal, motivation	Custom 5-point scale for nine emotions, SAM (valence, arousal, motivation)	43, 21/22, 22
[177]	2022	MMSD	ECG, PPG, EDA, EMG, GYRO	Shimmer sensing devices	SCWT, mental arithmetic, computer work, subtractions	Relaxation, stress, recovery	STAI-S, salivary cortisol (labeling) STAI-T, PSS4 (interpretation)	74, 38/36, 34
[178]	2022	SAM 40	EEG	Emotiv Epoc Flex (32-channel EEG gel kit)	SCWT, mental arithmetic tasks, mirror image recognition task	Task-induced short-term stress, relaxation	Stress rating scale (1–10)	40, 14/26, 21
[179]	2022	VERBIO	EDA, BVP, ECG, ST, ACC, speech signals	Empatica E4, chest-based Actiwave Cardio Monitor, Creative lavalier microphone	Real-life and VR public speaking tasks	Public speaking anxiety	STAI-T, CAI, PRPSA, BFI, BFNE, RWTC	55, 23/32, 22
[180]	2023	XR4DRAMA Stress Dataset	ECG, RSP, IMU, simulated emergency dialogs	Smart vest (ECG, RSP, IMU)	SCWT, cold pressor, stair climb, mental arithmetic, relaxation tasks	Stress	Stress self-annotation (0–100 scale per task)	5, 2/3, 22
[181]	2023	StressID	ECG, EDA, Resp, facial video, audio	BioSignalsPlux, Logitech QuickCam Pro 9000	Guided breathing, emotional videos, SCWT, MAT, public speaking	Stress, relaxation, neutral, arousal, valence	Self-assessment perceived stress (0–10) and relaxation, SAM (valence, arousal), two discrete labels	65, 18/47, 29
[182]	2023	EMAP	EEG, ECG, ResVP	BrainVision actiCHamp EEG amplifier-active Ag/AgCl 64-electrodeS, PowerLab 16/35 amplifier	Video Clips (rated on valence/arousal)	Positive, negative, discrete emotions	Valence, arousal, linking, engagement, discrete emotions: anger, sadness, happiness, disgust, fear	145, 93/48, 22
[183]	2024	BESST	ECG, EDA, ST, ACC, facial video, audio	Empatica E4, Faros 180, Zoom H4n recorder, Panasonic HC-VX9805 camcorder	Reading span task, hand immersion task	Labeling based on paradigm context and relaxation	PSS-14, STAI-Y1, NASA-TLX	90, 21/69, 19–26
[184]	2024	WorkStress3D	EDA, BVP, ST, ACC	Empatica E4, smartphone’s camera and microphone	Naturally occurring workplace stress (experience sampling)	Stress, mood, emotion	PANAS, general stress test, instant mood surveys	20, 35%/65, 38
[185]	2024	DSRP	EEG, HR	Neuroscan EEG cap, Huawei wrist HR monitor, PICO VR headset	Virtual reality scenarios (nature and animals)	Valence, arousal, dominance	SAM (arousal, valence, dominance), WHOQOL-BREF	15, N//A
[186]	2024	EEVR	EDA, PPG	Biopac MP36 (SS57LA and SS4LA modules), Meta Quest Pro VR headset	360° VR videos (eight from Russell’s circumplex quadrants)	Valence, arousal, dominance, PANAS emotions	SAM, PANAS, BFI-10, GHQ-12, qualitative self-reports	37, 16/21, 23
[187]	2024	EmoPairCompete	HR, EDA, BVP, ST, ACC	Empatica E4 wristband	Competitive tangram teamwork task (puzzle-solving in pairs)	Frustration, 10 PANAS emotions	I-PANAS-SF, visual analog scales (0–10)	28, N/A, 20–42
[188]	2024	MGEED	EEG, ECG, and OMG signals	Emotiv EPOC EEG 14 ch. Garmin HRM soft strap, Emteq smart glasses, Kinect	Emotional video clips	Positive, negative, discrete emotions	SAM (valence, arousal), Choice of happy, surprise, neutral, disgust, anxiety, sad, and fear	17, N/A, 18–40

### 3.1. Experimental Environments: Laboratory, VR, and Real-World Settings

Environmental settings of the experimental protocols span from controlled laboratory settings to immersive virtual reality environments, and to real-world settings, with each approach offering distinct value to the domain. Studies in laboratory settings dominate the landscape (only two in real-world settings and four in the lab incorporating VR), allowing researchers to use standardized stimuli to follow up the experimental process and therefore manage external factors of influence such as lighting, environmental temperature, noise, distractions, etc., enable the controlled and observable labeling of data, and ensure better signal quality due to reduced environmental noise. In contrast, datasets captured in real-world settings, such as NURSE, which include physiological signals of nurses working in real-world hospital settings during the COVID-19 outbreak, and WorkStress3D, which includes physiological signals, facial expressions, and speech signals, collected in real-world office environments with real work stressors, provide unique and spontaneous physiological and behavioral responses in natural environments. While real-world datasets enhance generalizability, challenges arise such as synchronization and labeling accuracy, noise control, and participants’ subjective bias. Other datasets incorporate VR settings that have become increasingly prevalent in affective research in recent years, such as VREED, VERBIO, DSRP, and EEVR. Although VR is expected to enhance emotional engagement, it comes with several challenges including difficulties in eliciting genuine emotions, accurate data annotation, adapting emotional models to VR scenarios, addressing individual and task variability, and effectively integrating multimodal data [189].

Following the experimental environment, attention shifts to the type of stimulus that can be employed, as the characteristics of the stimuli determine the reliability, validity, and comparability of physiological responses across studies.

### 3.2. Type of Stimulus

To elicit affective responses, a wide range and combination of stimuli are employed in the reviewed datasets, including emotional videos and images, mental and workload tasks, and social, physical, and real-life work-related stressors. Emotionally charged videos comprise the main approach on DEAP, MAHNOB-HCI, SEED, DREAMER, EEGEmotionDB, AMIGOS, Anxiety Dataset, Emognition, EMAP, and MGEED datasets. Videos are also included in datasets that use combinations of different stimuli such as WESAD, CLAS, PASS, and StressID. Video and picture stimuli require careful consideration of factors like duration (short clips < 6 s may not allow complex emotions to rise; long clips may result in passive responses), content realism (real-life content produces stronger responses especially for negative emotions), familiarity (reduce the emotional impact), and measurement methods to ensure reliable and valid results [190]. Datasets that include videos also incorporate self-reports of valence/arousal, and/or positive/negative emotion, and/or discrete emotion labeling, among others. However, inducing significant positive affect from positively charged videos or images has been challenging [190]. The challenge of distinguishing amusement from baseline physiological responses has also been highlighted in the WESAD dataset study (TSST to elicit stress, video to elicit amusement, and guided meditation for neutral conditions), which has stood out as a benchmark due to its experimental design and documentation [99]. Videos remain key stimuli for emotion classification and serve as indirect indicators of stress when linked with high arousal/low valence content. Nonetheless, it is evidenced that stimuli that combine social-evaluative threat and uncontrollability in motivated performance tasks are more effective in eliciting stress responses [191], such as the TSST [114]. An important number of datasets (SWELL-KW, STEW, CLAS, EEGMAT, SAM40, IDEA, MMSD, XR4DRAMA, StressID) employ, among others, mental or cognitive workload tasks, such as SCWT, MAT, and SIMKAP, to induce stress, relying on the interplay between performance, attention, and physiological arousal. WESAD and MuSe2021 (Ulm-TSST) employ the TSST, validated for stress response, which includes both social and cognitive aspects of stress, while BESST targets stress response via RST and the physical strain of the hand immersion task. Based on the principles of exposure therapy, DASPS use as stimuli the internal recall of real-life anxiety inducing events such as loss, trauma, and financial stress. DEAR-MULSEMEDIA explores multisensory multimedia approaches that integrate olfactory, haptic, and thermal cues with audiovisual stimuli to simulate realistic environments. EmoPairCompete mimics real environments by capturing social stress and frustration, using puzzle-solving in pairs of participants. XR4DRAMA merges laboratory protocols with tests in emergency field scenarios. Datasets that involve VR environments use stimuli such as immersive 360° environments (VREED), VR public speaking (VERBIO), VR scenarios of nature and animals (DSRP), and 360° VR videos (EEVR).

A wide range of elicitation methods exist which are further combined in respect to the research objective of each study. The inter-individual variability in physiological responses, with some participants experiencing strong physiological responses while others remain physiologically neutral despite self-reported affective changes, and the probable elicitation of mixed rather than discrete emotion states, contribute to the challenging task of mapping physiological responses to specific affective dimensions. Moreover, the stress induction method alters differently the degree of the activation of the stress mechanism, modulating physiological patterns. Multimodal approaches provide a comprehensive approach to capturing these physiological changes, while EEG approaches provide deeper insight into the underlying neurophysiological mechanisms and cognitive/emotional processing.

### 3.3. Multimodal and EEG Focused Approaches

Multimodal approaches are the most prevalent in the reviewed datasets, encompassing various physiological measures such as EEG, ECG, EDA, respiration, OMG, and ST, and in several cases combined with behavioral measures and contextual information such as facial expressions, speech, eye tracking and motion data, computer dialogs, and mouse and keyboard activity. The datasets exploit several wearable and fixed sensing systems, including the Empatica E4 wristband and Shimmer sensors for EDA, ECG, PPG, and temperature; RespiBAN, Faros 180, Garmin and BioHarness chest straps, and smart vests for respiration and ECG monitoring; Kinect, RGB-D cameras, and smart glasses for body posture, facial expressions, and eye tracking data; and microphones for audio. In contrast, EEG focused datasets, namely, SEED, STEW, DASPS, EEGMAT, EEG EmotionDB, SAM 40, and IDEA, rely on the measurement of the brain’s electrical activity, to identify alterations in the patterns of brainwaves in response to stress, providing a deeper understanding of the underlying emotional and cognitive processes. The datasets included EEG recordings which employed systems such as Biosemi ActiveTwo, NeuroScan, Emotiv EPOC, Clarity BrainTech, and Muse headsets, varying from high-density research grade equipment (32–64 channels) to lightweight wearable consumer devices with 4–14 electrodes (in most multimodal approaches). EEG focused approaches face signal fidelity challenges, require high spatial resolution and channel configuration, are sensitive to noise and artifacts from movement and muscle activity, and confront high computational complexity. Despite its challenges, EEG remains one of the most valuable and widely used modalities for stress analysis and affective computing, capturing rapid brain responses to stimuli, and offering a deeper understanding of stress response and the underlying mechanisms [87,192,193].

The variability in sensing systems employed to capture physiological signals that expand in variations such as sensor sensitivity, sampling rates, electrode placement, filtering, and data synchronization methods induce several limitations in comparability and cross dataset generalization of the developed models.

### 3.4. Data Synchronization

Multimodal approaches present the challenge of data synchronization from the multiple sensing devices. A later level of synchronization involves the temporal alignment of affective state annotations with the different experimental blocks. Across the thirty-two datasets, different synchronization methods are deployed, depending on the sensing systems, signal modalities, and experimental design. However, temporal coherence is the key factor that defines synchronization in all methods applied. Approaches include synchronization based on timestamp, device specific, software, extreme signal peaks, signal resampling, manual, real-time, and continuous annotation alignment.

Datasets that employ timestamp-based synchronization approaches use temporal markers or event triggers during the data acquisition process. DEAP employed a dedicated stimulus PC that sent synchronization markers directly to the recording PC aligning EEG, peripheral physiological signals, and frontal face video, while MAHNOB-HCI achieved synchronization through a central monitoring system combining a MOTU 8pre audio interface, camera trigger signals, and timestamps. AMIGOS used a PC for stimulus presentation, signal storage, and participants’ replies. Timestamp-based synchronization was also applied on XR4DRAMA Stress, StressID (using event annotation in OpenSignals), and MGEED (segmenting recorded time into non-overlapping windows of 0.5 s) datasets. In WorkStress3D, physiological signals, facial expressions, and audio recordings were aligned with the timestamps of the instant surveys. While in DEAR-MULSEMEDIA, it is reported that manual synchronization with content timestamps is performed. Synchronization based on a specific device was applied in BESST, where the Empatica E4 served as reference clock for aligning recordings from Faros 180, and manually handled signal drifts. Similarly, in WESAD, synchronization between the chest-worn RespiBAN and wrist-worn Empatica E4 was manually achieved through a double-tap gesture on the chest electrodes producing extreme signal peaks at the beginning and end of each recording. In the Emognition dataset, extreme accelerometer signals peaks from all devices were used for synchronization. A different approach was used in CLAS with a custom-built software application that aligned physiological signals, accelerometer data and the stimuli displayed on the PC. For the Ulm-TSST dataset, continuous annotation alignment was achieved by synchronizing audio, visual, text, and physiological signals to the label timestamps (visual features sampled at 2 Hz, matching the annotation rate, while text features aligned using timestamps obtained through the Montreal Forced Aligner). Co-registered event markers across EEG, peripheral, and behavioral data to maintain alignment throughout dynamic video-based tasks were used in the EMAP dataset. Unimodal approaches and datasets employing one sensing device required no synchronization, while VERBIO and DSRP do not provide details.

Data synchronization remains challenging due to differences in sampling rates, latency across devices, and temporal drift, which affect the precise alignment of signals and annotations. Regardless of the synchronization method applied, all datasets share the same objective that the physiological, behavioral, and contextual signals are temporally consistent and accurately aligned with the presented stimuli, enabling valid interpretation of emotional and stress responses, as the foundation for reliable analysis and model development.

### 3.5. Ground Truth

Ground truth labeling across the reviewed datasets is primarily defined by the experimental design, with the participants’ self-reports, external annotations, or performance-based metrics to be complementary sources of validation. Most laboratory studies adopt the dimensional model of affect, valence/arousal, or the PAD model, which are typically implemented through SAM. Other studies apply discrete emotion labeling through self-reported ratings or positive/neutral/negative classifications. Stress focused studies use validated stress inducing tasks combined with standardized questionnaires such as PANAS, STAI, NASA-TLX, PSS, and HAM-A, or, in the case of the MMSD dataset, combined with salivary cortisol sampling. Workload self-assessment ratings and task performance metrics are also used to infer cognitive strain. Other experimental frameworks employ continuous labeling to capture temporal affective changes, while in MuSe (Ulm-TSST), external raters provided independent annotations. Real-world datasets, such as NURSE, employed post shift survey validation of detected events and stress-related questionnaires, while WorkStress3D conducted semi-randomly six times daily over seven days instant stress-related surveys.

Overall, substantial variability exists in the applied methods for validating ground truth, ranging from self-reports and task-based to continuous or retrospective labeling. To reduce subjective bias, hybrid approaches that combine self-assessments with external annotations and biometric validation would enhance the objectivity and reliability of affective states’ ground truth.

### 3.6. Participant Demographics

Participant demographics differ in sample size, sex balance, and age range, following the experimental contexts of each study (Table 3). The number of participants ranges from 5 (XR4DRAMA Stress Dataset) to 145 (EMAP), with a median sample size of about 30–40 participants for most laboratory-based datasets. Nine studies include almost equal sex representation, while twelve studies overrepresent male participants and seven female participants. Most studies include participants with an age range of 18–30 years old, while only three studies have an age mean or span above 30 years old. Alarmingly, not all studies describe the sex distribution, the age span, and the mean age of participants.

Partial inconsistency can be found across the literature in regard to differences in autonomic responses and sex. Kelly et al. [194] reported that cortisol reactivity and autonomic arousal (elaborating TSST and ECG recordings) did not significantly differ among males and females, even though females reported higher levels of perceived negative affect than males, while other studies report that males tend to exhibit greater acute HPA and autonomic responses compared to women when facing the psychological stressors of public speaking [195]. Mikneviciute et al. [50] highlighted the necessity of further research on the interplay between sex and age in stress response and on the need for linking the psychological attributes of individuals to the physiological responses relevant to age and called for a validated age-related version of the TSST. Overlooking sex and age obstructs the development of generalized models for stress and emotion detection, especially when aiming for real-world settings. Even if a study does not explicitly focus on analyzing these differences, sex and age should be recorded, particularly for open benchmarking. Regarding ethnicity, most datasets do not provide such information.

The datasets predominantly represent healthy, young adults, with an overrepresentation of males, which constrains generalizability. Expanding demographic variability and ensuring balanced sex and age representation are essential, while the inclusion of social and economic factors would further enhance the validity and real-world applicability.

### 3.7. Class Imbalance

Class imbalance refers to the uneven distribution of samples among the different classes in a dataset, leading to a crucial methodological challenge, as models tend to be biased toward the majority class resulting in poor performance on the minority class, which is often the class of main interest, i.e., stress.

Class imbalance is profound also in the reviewed datasets with neutral or baselines affective states to be the majority class, such as in MANHOB-HCI, WESAD, and MGEED, or imbalances on valence and arousal annotations, such as in AMIGOS and EEVR. From a conceptual point of view, imbalance between normal and negative or high-arousal affective states is an expected and realistic reflection of everyday emotional experiences, as stress or intense emotional responses naturally occur less frequently than neutral or baseline states. This is met in the cases of the real-life datasets, NURSE and WorkStresd3D, where no stress classes constitute the majority classes. Ιn laboratory studies, the class balance of a dataset largely depends on the experimental design by ensuring the equal representation of affective states and baseline. Still, this approach allows only a moderate level of control, as the actual manifestation of emotions is not strictly uniform and remains highly dependent on individual differences and the intrinsic nature of the eliciting stimulus. For instance, in the WESAD dataset, the video stimuli of the amusement condition did not elicit particularly strong physiological responses, whereas in the DASPS dataset, the recall of traumatic experiences resulted in a higher number of samples in the severe anxiety class compared to the classes of lower anxiety levels. This contrast underscores how the experimental setup and stimulus selection can significantly shape class distribution. Moreover, ethical considerations impose additional constraints on the elicitation of negative emotions, particularly in inducing stress or anxiety for extended periods to balance with positive or neutral classes. To access the robustness of models under the challenge of class imbalance, DEAP, WESAD, and AMIGOS employed F1-score and leave-one-subject-out cross-validation as evaluation methods, while VREED and MAHNOB-HCI applied stratified k-fold cross-validation and feature selection based on ANOVA, respectively, to enhance the representation of different classes. In DASPS, to reduce the effect of unbalance, classes were regrouped, merging normal and light into one, and moderate and severe anxiety levels in a second class. To address class imbalance, StressID applied the synthetic data generation technique of SMOTE, EEVR performed oversampling, while MGEED compared the oversampling technique with the weighted loss function and class weighting and concluded that oversampling was more effective.

Despite the importance of balanced class distributions, not all datasets report details. Evaluation metrics and statistical analyses often provide a more accurate reflection of models’ performance, offering indirect insights of imbalance. Meanwhile, techniques such as synthetic data generation and oversampling are actively addressing the issue. Advances in balancing techniques and comparative evaluations of their impact on model performance would be valuable contributions; however, further research is also needed on how these techniques lead to problems such as overfitting or loss of information [196].

### 3.8. Accessibility and FAIRification

All datasets included in this review are available for research and educational purposes through various distribution channels, ranging from fully open access repositories to licensed academic agreements. Details of accessibility can be found in the cited study of each dataset presented in Table 3. Regarding Findability, Accessibility, Interoperability, and Reusability (FAIR) principles [197], none of the reviewed datasets explicitly state adherence to FAIR principles in their release study. Nevertheless, most demonstrate implicit alignment through open repositories, standardized formats, and detailed metadata. While the availability of open access datasets has improved in later years, consistent adherence to FAIR principles remains a critical area for further standardization.

### 3.9. Evaluation of Open Datasets

The evolution of the open access affective datasets in the last decade reflects the strong research interest, while their heterogeneity highlights the various approaches used to study the complex nature of stress and emotions. The reviewed datasets encompass a broad range of physiological signals, including EEG, ECG, EDA, and Resp, and of motion, audiovisual, and eye tracking data, while in most cases those are combined with standardized assessment questionnaires regarding individuals’ affective state. Experimental settings span from controlled laboratory experiments to real-world scenarios, such as the workplace, and to virtual reality environments. There is no ideal benchmark dataset for stress analysis. Each of the reviewed datasets follows an adequate design, providing a different dynamic in stress research and serving distinct methodological and application needs.

In laboratory settings, WESAD and MMSD are the strongest reference datasets due to their experimental precision and validated stress induction protocols, making them effective for algorithm benchmarking, feature analysis, and multimodal model development. WESAD has become the standard for wearable stress detection as it elaborates the TSST despite the small and sex unbalanced sample size, while MMSD uniquely provides validation through salivary cortisol and a sufficient and sex balanced sample size. Although WESAD and MMSD enable multimodal approaches, they do not incorporate EEG recordings, which are critical for the in-depth analysis of cognitive and emotional processes. SAM40 and DASPS stand out in the analysis of neural mechanisms of stress. SAM40 provides 32-channel EEG using a combination of controlled cognitive stressors from a sufficient sample size but is sex unbalanced, while DASPS uniquely use the recall of real-life anxiety inducing events and captures EEG using a 14-channel headset, from an acceptable and almost sex balanced sample size. In addition, EEGmat and STEW further enable the analysis of cognitive and mental workload, with EEGmat to include EEG recordings mainly from females and STEW only from males. However, these datasets enable only unimodal approaches. DEAP, MAHNOB-HCI, SEED, WESAD, AMIGOS, MMSD, SAM40, STRESSID, and EMAP are the most methodologically balanced open datasets, in terms of protocol design, stimuli, signal fidelity, and sample size, making them the most suitable for exploring different approaches in model development.

In contrast, NURSE and WorkStress3D lack the laboratory precision but capture the physiological responses of real-life occupational stress, making them suitable for developing and testing real-world, context-aware stress detection models that can be generalized beyond experimental conditions. Finally, VR-based datasets such as VREED, DSRP, and EEVR offer a foundation for exploring laboratory precision with VR and immersive realism.

Nevertheless, limitations and challenges remain, including imbalanced representation of affective states, validation of the effectiveness of the induction method, variability on labeling methods, limited demographic representation with an underrepresentation of women and predominance of young adults, and variable signal quality, particularly for EEG, the processing of which requires high-fidelity recordings. To mitigate these challenges, ongoing efforts in data preprocessing, signal enhancement, and class balancing are deployed to support the development of more robust models and enhance their generalizability.

## 4. Current Trends in Stress Research

The methodological landscape of stress analysis using physiological signals has significantly evolved in recent years [198]. To reflect this evolution, a small and comprehensive review of the methodological approaches applied in the last three years (2023–2025) for stress assessment is reported. The key experimental and computational details such as the datasets used, signal modalities, preprocessing and feature extraction techniques, modeling approaches, evaluation methods, and reported results are presented in Table 4. Current research trends reflect the intersection of advanced sensing technologies, machine learning, and deep learning algorithms [199].

**Figure 3:**
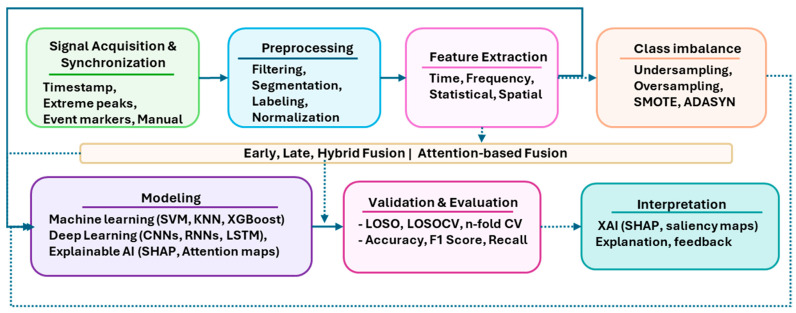
Conceptual framework of the processing pipeline for stress detection based on physiological signals.

Multimodal data fusion is the most prominent method to capture complementary aspects of the stress response by deploying early, late, or hybrid fusion techniques. In early fusion, raw or preprocessed physiological signals are concatenating before feature extraction, enabling the model to learn complex interactions and complementary information, but it requires well synchronized data of consistent size and dimensions, high computational resources, and may lead to overfitting especially in small datasets. In late fusion, independent classifiers are deployed for each signal, preserving modality-specific features, and then later ensemble at a decision level, offering flexibility in asynchronized and different dimensions and size of data, and requires less computational complexity, but may miss interactions and dependencies between modalities. In hybrid fusion, the advantages of both early and late techniques are combined, leveraging raw data interactions and modality-specific predictions, with high computational cost, complexity in design, and the need for large datasets [200,201]. Key physiological signals that are combined to improve classification accuracy and robustness are the EEG, ECG, EDA, PPG, and respiration [202,203], with EEG to outstand on performance and therefore act as a standalone signal on models [203,204]. Machine learning continues to play a foundational role, with traditional models like SVMs, Random Forests, and XGBoost still used in settings with limited or well-structured features [202,205,206]. Deep learning and representation learning has gained major research interest, exploring CNNs, recurrent models (LSTM/Gated Recurrent Units (GRUs)), time series transformers and self-supervised learning methods to pretrain models without labels, shifting from handcrafted features towards automatically learned features from raw signals [199,201]. Personalized and adaptive models are also utilized to handle the variability of stress responses across individuals by setting personal baselines of stress, and by using minimum data for individual learning and transfer learning techniques where pretrained models are adapted to new individuals [203]. A schematic representation of the conceptual framework of the processing pipeline for stress detection based on physiological signals can be found in Figure 3.

A machine learning framework for multi-class anxiety detection using ECG, EDA, and a range of contextual features exploiting the WESAD dataset was presented by Jain and Kumar [205]. The physiological signals were preprocessed, segmented, and transformed into statistical and domain-specific features, while contextual data included lifestyle and demographic attributes. The six-item STAI questionnaire was used to derive three class (low, moderate, high) anxiety labels. To address class imbalance, multiple oversampling techniques, such as Synthetic Minority Oversampling Technique (SMOTE), borderline SMOTE, adaptive synthetic sampling approach for imbalanced learning (ADASYN), and random oversampling, were tested. The best results were obtained using Gradient Boosted Decision Trees (GBDTs) with leave-one-out cross-validation (LOOCV), achieving an accuracy of 97.3% when using both physiological and context features, and random oversampling.

Using fusion techniques of EEG and peripheral physiological signals, Pei et al. [207] presented a machine learning approach for the detection of social and mental stress at different levels. Eleven participants underwent the MIST and TSST stress-inducing protocols, while EEG, PPG, HR, EDA, and temperature data were collected. The signals were segmented into various time windows of 60, 30, 10, and 4 s, and a wide range of features were extracted. The EEG signals were decomposed using discrete stationary wavelet transform (DSWT), followed by independent component analysis (ICA) to remove ocular artifacts, and then reconstructed using inversive DSWT. A comparative study of LDA, SVM, KNN, Decision Tree (DT), and Naïve Bayes (NB) machine learning algorithms was performed. Classification accuracy of 99.8% was achieved for EEG signals when using LDA with a 60 s window, and 98.3% for peripheral signals using DT and a 4 s window. The study demonstrates the promise of short-duration, multimodal stress detection with potential for real-time applications.

A deep learning framework with a double early fusion strategy of biosignals, speech, and facial expressions was presented in the first introduction of the WorkStress3D dataset by Dogan et al. [201]. The multimodal CNN-based model was tested on both single and fused modalities. The early fusion model outperformed transfer learning (fusion of facial expressions and audio followed by biosignals) and modality-specific models, reaching an F1 score of 0.94 with a loss of 0.18, with authors to highlight the difficulty of adapting pre-trained models (on image and audio datasets). Comparison tests revealed that the non-sequential Deep Neural Network (DNN) outperformed the sequential DNN, sequential LSTM, non-sequential Bidirectional LSTM, and sequential GRU models.

A fuzzy ensemble deep learning model for EEG-based emotion classification across the valence and arousal dimensions was proposed by Dhara et al. [199]. Authors combined three deep learning models (CNN/LSTM, CNN/GRU, and 1D-CNN) using a Gompertz function-based fuzzy rank fusion method. The approach was validated on DEAP and AMIGOS datasets in both subject-dependent and subject-independent settings, achieving up to 98.66% and 99.38% accuracies, respectively. The study emphasizes ensemble reliability and performance consistency across datasets and training strategies.

**Table 4:** Overview of methodological approaches in stress detection (2023–2025).

Ref. (Year)	Dataset Used	Signal Modalities	Feature Extraction	Modeling Approach	Evaluation Protocol	Key Results (Accuracy %)
[205] (2024)	WESAD	ECG, EDA, and context features	ECG: time and statistical; EDA: SCR peak magnitude/duration and statistical; Context: BMI, caffeine, exercise, posture, etc.	DT, GBDT with multiple balancing methods	LOOCV with imbalanced data handling (SMOTE, ADASYN)	GBDT and LOOCV: 97.3% (ECG, EDA, context), ECG only: 89.8%, EDA only: 85.9%
[207] (2024)	Internal dataset (11 subjects, TSST, MIST)	EEG, PPG, HR, Temp, SpO_2_, EDA	Time, frequency, geometric domain (EDA, HRV, EEG power, and ratios), EEG artifact removal (ICA and DSWT)	LDA, SVM, KNN, DT, NB	10-fold CV, windows (60, 30, 10, 4 s), within-subject classification	Stress type (LDA, 60 s): 99.8%; Stress level (DT, 4 s): 97.8%; EEG alone best at long windows, physio best at short
[201] (2023)	WorkStress3D and own dataset (daily workplace)	EDA, BVP, ST, ACC, audio, facial video	Biosignals: down sampling, normalization, polynomial transformation; Audio: Mel-spectrogram-MFCCs, Face: CNN preprocessing alignment	CNN-based early fusion model, modality-specific subnetworks, transfer learning ResNet/VGG16)	80/20 split and 10-fold CV, (15 s, 30 s, 60 s) windows	Early fusion F1: 0.94, transfer learning F1: 0.93, best accuracy: 94%
[199] (2024)	DEAP, AMIGOS	EEG (14 channels)	FFT-based frequency features in 5 bands, segmented 2 s	Ensemble DL (CNN/LSTM, CNN/GRU, CNN), fuzzy Gompertz function	Subject-independent (60/20/20 split), subject-dependent (per-subject average)	DEAP: 95.97%, AMIGOS: 99.38%
[204] (2024)	DEAP, SEED	EEG	Differential entropy (DE) across frequency bands, time windowed	STLGCNN: Attention/BiLSTM/GCNN/LSTM	10-fold CV, subject-dependent	DEAP: 94.16%, SEED: 96.78%
[208] (2024)	DREAMER, SEED, SEED-IV,	EEG	Time (Hjorth), frequency domain (α, β, δ), band ratios, frontal asymmetry, CSP maps	MLP (2 hidden layers), ReLU, dropout	LOTO (trial), LOSO (subject), 10-fold CV	DREAMER: 94%, SEED-IV: 44% SEED: 62%
[209] (2024)	SEED, SEED-V	EEG	STFT-DE features, sliding window composition (SWC), spatial and temporal encoding	Static spatial adapter, temporal causal network (GRU and MHA)	Subject-independent (8/2 split), ablation and cross-subject testing	SEED: 95.35% SEED-V: 94.28%
[210] (2024)	WESAD	ECG, EDA, EMG, BVP	Bandpass, normalized, windowed, attention maps/CNN/LSTM	CNN–LSTM with feature-level and semantic-level attention fusion	5-fold cross-validation	Accuracy 83.88%, F1: 0.85
[211] (2023)	Internal dataset (24 subjects, MAST-CPT, MAT)	ECG, EDA, EOG, EEG	Time, frequency, and statistical features, interpretable feature subset	Classical ML (SVM, RF, XGBoost, LDA) and SHAP XAI	Leave-three-out nested CV, SFFS feature selection, SHAP explanation	86.5% balanced accuracy (XGBoost, 45 s, full fusion), 81.5% interpretable features
[212] (2024)	Internal dataset (28 subjects, airhorn stimulus)	EDA, ST	Raw EDA and ST, preprocessed phasic signals (1 Hz, 16 s sliding windows)	LSTM ensemble, conditional GAN, integrated gradients	3 train-test seeds, 5-fold CV grid search, time-windowed evaluation	LSTM-DGE: Recall: 76.3%, Precision: 35.9%, Accuracy: 98.1%, Rule-based: Recall 73.3%, Precision 32.3%
[213] (2025)	WESAD	EDA, PPG, Temp, ACC	Raw signals, human-engineered features, sliding window, normalization	Residual attention DNN with multi-head blocks, Guided Grad-CAM for explainability	LOSO CV	Stress: 96.57%, Emotion: 87.77%
[214] (2024)	SWEET (240 subjects, free-living context, wearables)	ECG, ST, skin conductance	Time and statistical features, 3-class and binary labels, SMOTE for balancing	Classical ML: RF, XGBoost, SVC, KNN, DT	4 settings: binary and 3-class, with and without SMOTE	Binary: RF 98.29%, 3-class: XGBoost 98.98%
[215] (2025)	Nurse Stress Prediction	EDA, HR, skin temp, ACC	Time domain, statistical, FFT spectral features, sliding window, jittering, 60 s windows segments	Dual-branch CNN (time, frequency), FC classifier	Stratified split 80/20, hyperparameter tuning via Bayesian optimization	Own model: 91%, outperformed RF, SVM, XGBoost

To address the instability of emotion intensity and underutilization of EEG biotopological information, Xu et al. [204] proposed a Spatio-Temporal Learning Graph Convolutional Neural Network (STLGCNN) that integrates an attention module, BiLSTM, a graph convolutional neural network (GCNN), and an LSTM module. The model uses an attention mechanism to identify correlations between different time periods and to reduce emotional temporal volatility while the GCNN learns the bio-topological information of multi-channel EEG signals and extracts effective graph domain features, which are then fed into the LSTM to integrate the graph-domain information and extract valid temporal information. The approach was evaluated on the DEAP and SEED datasets and achieved average accuracies of 93.81% and 96.78%, respectively.

A combination of time-domain (Hjorth parameters), frequency-domain (Frontal asymmetry), and topographical maps (Common Spatial Patterns (CSPs)) EEG features, and a multi-layer perceptron (MLP) classifier, was proposed by Giannakakis et al. [208] to explore EEG-based emotion recognition. The method was validated on three publicly available datasets, DREAMER, SEED, and SEED-IV, and assessed by both subject-dependent, using leave-one-trial-out (LOTO), and subject-independent, exploring leave-one-subject-out (LOSO). The model achieved an accuracy up to 94% in predicting arousal and 93% valence for DREAMER, while moderate scores up to 62% and 44% were reported for SEED and SEED-IV datasets with multiple discrete emotional states.

Dong et al. [209] introduced a deep learning model for cross-subject EEG-based emotion recognition which achieved an accuracy of 95.35% on SEED and 94.28% on SEED-V, while it also demonstrated high efficiency in emotion classification. The architecture integrates a static spatial adapter to encode topological brain activity patterns, and a temporal causal network to capture the temporal dependencies and causal relationships between brain regions. The model was fed with differential entropy features extracted from Short-Time Fourier Transform (STFT) transformed EEG signals.

Using multimodal physiological signals from the WESAD dataset and a deep learning architecture that integrates a CNN–LSTM backbone with both feature-level and semantic-level attention mechanisms, an emotion classification of 83.88% accuracy was achieved in the study of Zou et al. [210]. The proposed CNN–LSTM–attention neural network boosted accuracy by 4.58% compared to baseline CNN–LSTM, while semantic-level attention particularly improved the classification of neutral and stress. In a further evaluation, authors made a comparison with the machine learning algorithms and accuracies: DT 58.6%, Random Forest (RF) 71.4%, LDA 79.3%, KNN 57.3%, and AdaBoost Decision Tree (ABDT) 80.3%.

Meanwhile, explainable AI (XAI) is being introduced into stress modeling pipelines to enhance the interpretation of physiological signal analysis methods. XAI is used to better explain and interpret the process of feature learning and decision making that leads to model predictions [198]. Techniques such as Shapley additive explanation (SHAP) values, attention mechanisms, and saliency maps provide insight into which features or time segments drive model decisions [198].

Tervonen et al. [211] investigated how different stressors produce distinct physiological stress patterns through mixed model statistical analysis and explainable machine learning classification. Physiological responses from 24 healthy participants were measured using MAST, comprising alternating trials of cold pressor and mental arithmetic, while ECG, EDA, EOG, and EEG signals were collected. Seven classical ML models were compared, kNN, LDA and quadratic discriminant analysis (QDA), SVM, DT, RF, and extreme gradient boosting (XGBoost), while SHAP values were used to interpret feature contributions to stress type prediction. Window sizes of 10, 15, 30, and 45 s were tested with results to show that longer windows tended to perform better regardless of classifier type and feature set. Feature selection was performed using sequential forward floating search (SFFS). In addition, a nested leave-three-out validation was conducted to select the features and the best hyperparameters. The best-balanced accuracy reached 86.5% with a 45 s window length and XGBoost.

To detect acute stress based on physiological recordings of 28 participants wearing an Empatica E4, Moser et al. [212] proposed an explainable deep learning methodology. The model integrated an LSTM network and extended through a Deep Generative Ensemble (DGE) of conditional generative adversarial networks (GANs). For explainability, integrated gradients (IGs) were leveraged to reveal features used by the model for prediction. The model achieved recall of 76.3% and precision of 35.9%, while it has also been compared to a previously published rule-based system.

An explainable deep learning architecture for stress and emotion detection using multimodal biosignals from the WESAD dataset was introduced by Lee et al. [213]. The model integrates raw signals of EDA, PPG, ST, and ACC with human-engineered features to improve both accuracy and interpretability. A residual attention mechanism was applied, and explanations were generated via guided gradient-weighted class activation mapping (Grad-CAM), highlighting channel-wise and temporal saliency. To evaluate the performance, the leave-one-subject-out cross-validation (LOSO) method was deployed. The model achieved 96.57% accuracy on binary stress classification, and 87.77% on three labels of emotion classifications. Furthermore, a survey with medical doctors verified that their proposed XAI method was more understandable than traditional saliency maps.

Researchers apply approaches to go beyond lab settings, into real-world settings, using wearable sensors to capture physiological data with contextual information. Alim et al. [214] proposed a machine learning framework for the detection of stress in free-living environments exploiting physiological signals from wearable sensors. The SWEET dataset [156] was utilized which includes ECG, skin temperature, and skin conductance data from 240 participants collected during natural daily activities in the work environment. The study evaluated five classical machine learning models, namely, K-Nearest Neighbors (KNN), Support Vector Classification (SVC), DT, RF, and XGBoost across binary and three-level stress classification, both with and without SMOTE oversampling. The best results were performed by Random Forest for binary classification without SMOTE reaching an accuracy of 98.29%, and by XGBoost for three-level classification with SMOTE reaching an accuracy of 98.98%, showing the effectiveness of classical models with proper preprocessing and imbalance handling.

Aiming to achieve stress detection in real-world settings, Xiang et al. [215] elaborated the NURSE dataset and proposed a multimodal deep learning framework that integrates time-domain and frequency-domain features from physiological recordings. Preprocessing of signals included 60 s windows with 50% overlap, normalization of min–max scaling, followed by 15 min threshold segmentation and data augmentation through sliding windows and Gaussian jittering. Class imbalance was addressed by applying SMOTE. The classification model consisted of parallel CNNs to process time-domain and frequency-domain features separately and then followed by fully connected layers for final classification. Comparison with baselines modes, including Logistic Regression (LR), Naïve Bayes, Random Forest, Decision Tree, KNN, AdaBoost, and XGBoost was conducted, with the proposed model to outperform by reaching an accuracy of 91%.

## 5. Discussion

An overview of stress analysis frameworks and methods based on physiological signals was presented. This work is subject to limitations that must be considered in the interpretation of the findings and results: (1) For the systematic review of open datasets, the selection and evaluation process was performed by a single reviewer, which may introduce subjective bias in the inclusion and exclusion process; (2) There was no preregistered protocol of the systematic review, which increases the risk of bias and the possibility of duplicated effort; (3) The search strategy of the systematic review included only the electronic academic databases of Scopus and Google Scholar and online research repositories, which may have limited the results; (4) Variations in the datasets’ designs, frameworks, and methodological approaches limited the comparability and critical evaluation of some findings; (5) As this review covers multiple aspects and dimensions of stress research, an in-depth analysis of each was not feasible, which may constrain the level of detail and precision; (6) No quantitative meta-analysis was performed and therefore conclusions should be interpreted as qualitative insights.

Stress is a complex and multifaceted phenomenon which has profound effects on both physiological and psychological systems. Although stress response varies between individuals, recognizable associated patterns can be captured via biosignals such as EEG, ECG, EDA, and Resp, which reflect physiological changes. The variability of stress responses arises from several interacting factors, including the nature, intensity, and duration of the stressor, as well as the individual’s perception of controllability and predictability [49,52,53]. Biological and psychosocial factors such as sex, age, genetics, personality traits, coping mechanisms, early life experiences, cultural background, and physical or mental health further modulate the stress response [49,53,54,55,150]. Differences in the neurobiological response to acute stress based on sex and age groups have been highlighted in the literature [114], leading to the need to address the issue of the underrepresentation of women and predominance of young adults in open datasets. Future datasets should expand demographic variability and ensure balanced sex, age, and cultural groups, including middle-aged and older adults, who remain underrepresented in most current studies. Furthermore, to account for individual variability in stress responses, psychological (e.g., SMM, EPQ, BFI-10) and lifestyle (e.g., sleep, smoking, medication, exercise) covariates should be incorporated [50].

Regarding the experimental framework settings, the stressor used, whether cognitive, emotional, or physical, influences the physiological response and the subsequent signal patterns. Stress induction methods which combine mental effort, social strain, and task performance pressure, such as TSST, significantly differ from methods such as emotionally charged images or videos [190,191]. Further, the content of the stimuli should be carefully adjusted to the age group and the characteristics of the participating population following a standardized process, to be effective [50]. The reliability of stimuli is critical, as it can lead to imprecise ground truth labels and in turn affect the robustness of models trained on such data. Establishing ground truth for affective states is another critical task. Self-reported assessments provide valuable insight into subjective experience but are influenced by introspective ability and subjective bias. Experimental setting-based labeling provides a more neutral baseline but presumes consistent emotional responses across individuals and stressors, while external observer annotations are limited on visible manifestations of emotions and are also prone to subjective bias. Consequently, the most promising approach for ground truth labeling combines structured experimental design, individual’s self-reporting, and physiological measures to ensure both objectivity and personal validity.

The systematic review, over the last decade, on open access affective datasets eligible for stress analysis based on physiological signals, reflects the growing research interest and the evolution of signal processing and modeling methods. Datasets incorporate various physiological signals, including EEG, ECG, and EDA, as well as behavioral measures such as motion, audiovisual, and eye tracking data, enabling multimodal and crossmodal approaches. Furthermore, experimental settings span from controlled laboratory experiments to virtual reality environments and to real-world scenarios such as the workplace. Nevertheless, challenges and limitations that deal with the sensing and processing of the signals remain, especially for in-depth analysis and benchmarking. Limited to the sensing capabilities of each device, further challenges include motion artifacts, environmental noise, variability in sensor placement and contact, processing techniques and signal fidelity, data synchronization and labeling, and class imbalance. To mitigate these challenges, ongoing efforts in data preprocessing and signal enhancement, such as windowing, filtering, normalization, artifact removal, and transformations, and class balancing techniques such as SMOTE [205,214], ADASYN [205], over/under-sampling, and ensemble methods [199,212], are deployed. The heterogeneity in experimental protocols, sensing systems and signals, and preprocessing techniques raises difficulty in performing comparative studies.

Current research directions focus on improving the robustness and interpretability of stress detection systems. Multimodal data fusion techniques, deep learning, transfer learning, and domain adaptation approaches are increasingly employed, exploring CNNs, LSTM/GRUs, time series transformers, and self-supervised learning methods, to address inter-individual variability and enable models to generalize across contexts and populations. Meanwhile, machine learning approaches continue to play a foundational role, with traditional models like LDA, SVM, KNN, DT, and NB to be deployed. Explainable AI methods such as saliency mapping and attention mechanisms are gaining attention to ensure transparency in decision making processes and to identify the physiological features that drive model decisions. Despite these advancements, several difficulties and challenges persist in developing robust and interpretable stress detection models. Multimodal fusion of physiological and behavioral data is challenged by addressing, feature redundancy, modality heterogeneity, and insufficient inter-modal supervision [216,217]. Deep learning and transfer learning models require large and balanced datasets, which are often lacking, leading to risks of overfitting and limited generalizability [218,219]. Explainable AI techniques offer transparency on the models’ decisions, but challenges remain in balancing performance and interpretability, handling high dimensional data, and establishing evaluation and validation methods [198].

## 6. Conclusions

In this work, a systematic review of open access affective computing datasets was conducted, focusing on those suitable for stress analysis based on physiological signals, outlining methodological frameworks, identifying current computational trends, and highlighting key challenges and future research directions. Physiological signals and current modeling techniques can enable a robust framework for stress assessment. However, no matter how advanced computational models become, the reliability of outcomes remains highly dependent on the quality of the input signals, the validity of the stress induction protocol, and the accuracy of ground truth labeling. A well-defined experimental framework that accounts for the dynamic interaction between individual characteristics, stressor properties, and the underlying physiological mechanisms of stress responses should integrate individual demographics, personality traits, and standardized self-assessment scales, along with clearly defined experimental protocols that minimize bias, and employ high-quality sensing systems. Moving towards real-world applicability requires bridging laboratory precision with in-the-wild sensing. Studies that perform parallel recordings with high end sensing equipment and wearables could reinforce this transition with comparative analysis and crossmodal validation. Ultimately, progress in sensor technology, experimental standardization, and computational advances will be key for the development of reproducible, interpretable, and generalizable models of stress and emotions. Future research should also explore innovative directions, such as federated learning, that enable the collaborative training of shared models, ensuring data privacy and security by keeping data local [220], and privacy-preserving data sharing which facilitates trustworthy data sharing [221]. These emerging approaches could enhance stress modeling and affective computing by enabling collaborative analysis across large-scale datasets while maintaining ethical and legal compliance with data protection regulations and participant confidentiality.

## Abbreviations

The following abbreviations are used in this manuscript:

ABDT	AdaBoost Decision Tree
ACC	Accelerometer
ACTH	Adrenocorticotropic Hormone
AdaBoost	Adaptive Boosting
ADASYN	Adaptive Synthetic Sampling Approach For Imbalanced Learning
AgCl	Silver Chloride
AI	Artificial Intelligence
ANOVA	Analysis of Variance
ANS	Autonomic Nervous System
API	Application Programming Interface
AUs	Action Units
BAI	Beck Anxiety Inventory
BESST	Brno Extended Stress and Speech Test
BFI	Big Five Inventory
BFNE	Brief Fear of Negative Evaluation
BiLSTM	Bidirectional Long Short-Term Memory
BORG	Borg Rating of Perceived Exertion
BVP	Blood Volume Pulse
CAI	Communication Anxiety Inventory
CAMCOG-R	Cambridge Cognitive Assessment (Revised)
CHIPS	Cohen–Hoberman Inventory of Physical Symptoms
CLSP	Contrastive Language Signal Pre-training
CNN	Convolutional Neural Network
CPT	Cold Pressor Test
CRH	Corticotropin-Releasing Hormone
CSP	Common Spatial Patterns
CV	Cross-Validation
DASS-21	Depression Anxiety Stress Scales
DBNs	Deep Belief Networks
DE	Differential Entropy
DGE	Deep Generative Ensemble
DL	Deep Learning
DNN	Deep Neural Network
DSI	Daily Stress Inventory
DSM-5	Diagnostic and Statistical Manual of Mental Disorders, Fifth Edition
DSWT	Discrete Stationary Wavelet Transform
DT	Decision Tree
DWT	Discrete Wavelet Transform
ECG	Electrocardiography
EDA	Electrodermal Activity
EEG	Electroencephalography
ELM	Extreme Learning Machine
EMG	Electromyography
EOG	Electrooculogram
EPQ	Eysenck Personality Questionnaire
ES	Experience Sampling
FFT	Fast Fourier Transform
GAD	Generalized Anxiety Disorder
GAN	Generative Adversarial Network
GBDT	Gradient Boosted Decision Tree
GCNN	Graph Convolutional Neural Network
GHQ-12	General Health Questionnaire-12
Grad-CAM	Gradient-Weighted Class Activation Mapping
GRUs	Gated Recurrent Units
GSR	Galvanic Skin Response
HAM-A	Hamilton Anxiety Rating Scale
HPA	Hypothalamic–Pituitary–Adrenal
HR	Heart Rate
HRV	Heart Rate Variability
IADS	International Affective Digitized Sounds
IAPS	International Affective Picture System
IBI	Interbeat Interval
ICA	Independent Component Analysis
IG	Integrated Gradients
IMU	Inertial Measurement Unit
KNN	K-Nearest Neighbor
LDA	Linear Discriminant Analysis
LOOCV	Leave-One-Out Cross-Validation
LOSO	Leave-One-Subject-Out
LOTO	Leave-One-Trial-Out
LR	Logistic Regression
LSTM	Long Short-Term Memory
MAST	Maastricht Acute Stress Test
MAT	Mental Arithmetic Task
MCA	Multimedia Content Analysis
MD-DE	Modified Differential Entropy
MFCCs	Mel-Frequency Cepstral Coefficients
MHA	Multi-Head Attention
MIST	Montreal Imaging Stress Task
ML	Machine Learning
MLP	Multi-Layer Perceptron
MMSE	Mini-Mental State Examination
MRI	Magnetic Resonance Imaging
NASA-TLX	NASA Task Load Index
NB	Naïve Bayes
OMG	Optomyography
PANAS	Positive and Negative Affect Schedule
PASAT	Paced Auditory Serial Addition Test
PCL-5	Post-Traumatic Stress Disorder Checklist
PFC	Prefrontal Cortex
PPG	Photoplethysmography
PQ	Presence Questionnaire
PRPSA	Personal Report of Public Speaking Anxiety
PSS	Perceived Stress Scale
PTCI	Post Trauma Cognitions Inventory
PTSD	Post-Traumatic Stress Disorder
QDA	Quadratic Discriminant Analysis
RBF	Radial Basis Function
ReLU	Rectified Linear unit
ResNet	Residual Network
Resp	Respiration
RF	Random Forest
RGB	Red, Green, and Blue
RSME	Rating Scale Mental Effort
RSPAN	Reading Span Task
RWTC	Reticence Willingness to Communicate
SAM axis	Sympathetic–Adreno–Medullary
SAM	Self-Assessment Manikin
SCR	Skin Conductance Response
SCWT	Stroop Color Word Test
SFFS	Sequential Forward Floating Search
SHAP	Shapley Additive Explanation
SIMKAP	Simultaneous Capacity
SMM	Stress Mindset Measure
SMOTE	Synthetic Minority Oversampling Technique
SRI	Stress Response Inventory
SSAE	Stacked Sparse Autoencoder
ST	Skin Temperature
STAI	State-Trait Anxiety Inventory
STFT	Short-Time Fourier Transform
STLGCNN	Spatio-Temporal Learning Graph Convolutional Neural Network
SVC	Support Vector Classification
SVM	Support Vector Machine
SWC	Sliding Window Composition
TSST	Trier Social Stress Test
TST	Thermal Stress Test
VAS	Visual Analog Scale
VR	Virtual Reality
WHOQOL-BREF	World Health Organization Quality of Life
XAI	Explainable Artificial Intelligence
XGBoost	Extreme Gradient Boosting
